# Fission Yeast Pxd1 Promotes Proper DNA Repair by Activating Rad16^XPF^ and Inhibiting Dna2

**DOI:** 10.1371/journal.pbio.1001946

**Published:** 2014-09-09

**Authors:** Jia-Min Zhang, Xiao-Man Liu, Yue-He Ding, Liang-Yao Xiong, Jing-Yi Ren, Zhi-Xiong Zhou, Hai-Tao Wang, Mei-Jun Zhang, Yang Yu, Meng-Qiu Dong, Li-Lin Du

**Affiliations:** 1 National Institute of Biological Sciences, Beijing, China; 2 Graduate School of Peking Union Medical College, Beijing, China; Mount Sinai Hospital, Canada

## Abstract

Structure-specific nucleases play crucial roles in many DNA repair pathways. They must be precisely controlled to ensure optimal repair outcomes; however, mechanisms of their regulation are not fully understood. Here, we report a fission yeast protein, Pxd1, that binds to and regulates two structure-specific nucleases: Rad16^XPF^-Swi10^ERCC1^ and Dna2-Cdc24. Strikingly, Pxd1 influences the activities of these two nucleases in opposite ways: It activates the 3′ endonuclease activity of Rad16-Swi10 but inhibits the RPA-mediated activation of the 5′ endonuclease activity of Dna2. Pxd1 is required for Rad16-Swi10 to function in single-strand annealing, mating-type switching, and the removal of Top1-DNA adducts. Meanwhile, Pxd1 attenuates DNA end resection mediated by the Rqh1-Dna2 pathway. Disabling the Dna2-inhibitory activity of Pxd1 results in enhanced use of a break-distal repeat sequence in single-strand annealing and a greater loss of genetic information. We propose that Pxd1 promotes proper DNA repair by differentially regulating two structure-specific nucleases.

## Abstract 2

During DNA double-strand break repair, two structure-specific DNA nucleases are controlled by the same regulator Pxd1, but in opposite manners.

## Author Summary

Genome stability maintenance relies on DNA repair enzymes, among which are structure-specific nucleases that cleave DNA in a sequence-independent but structure-dependent manner. It is important to understand how the activities of such nucleases are controlled, because either insufficient or excessive cleavage of DNA could jeopardize genome integrity. In this study, we discovered a new regulator of two different structure-specific nucleases in the fission yeast *Schizosaccharomyces pombe*. The identified protein, which we named Pxd1, promotes the activity of the 3′ endonuclease Rad16, but restrains the activity of the 5′ endonuclease Dna2. In the absence of Pxd1, several Rad16-dependent DNA repair processes become defective. One of these processes is a DNA-repeat–mediated double-strand break repair pathway called single-strand annealing, which causes genomic deletions. When the Dna2-inhibitory activity of Pxd1 is impaired, Dna2-dependent end processing of double-strand breaks is enhanced and a more extensive deletion occurs during single-strand annealing. Thus, Pxd1 facilitates a potentially dangerous DNA repair process, but in the meantime minimizes its deleterious consequences. We propose that a dual-target regulator like Pxd1 is ideally suited for coordinating multiple enzymatic activities during DNA repair.

## Introduction

Structure-specific DNA nucleases contribute to the maintenance of genome stability by processing DNA secondary structures during DNA replication and repair [1],[2]. The activities of these nucleases must be tightly controlled to prevent unintended cleavage; however, the molecular mechanisms underlying the regulation of these nucleases have not been fully elucidated.

The roles of several structure-specific nucleases in DNA repair are best understood in the single-strand annealing (SSA) pathway of DNA double-strand break (DSB) repair. SSA is a repair pathway for DSBs occurring between repeat sequences and has been most thoroughly studied in the budding yeast *Saccharomyces cerevisiae* [3]. SSA relies on the DNA resection process to generate 3′-ended single-stranded DNA (ssDNA) extending from the break to the repeat sequences [4]. Such long-range resection is mediated by two structure-specific nucleases, Exo1 and Dna2, which act in parallel to each other [5]. Upon annealing of the ssDNA of the repeat sequences, the intervening sequence between the repeats, which now becomes 3′ nonhomologous ssDNA tails, is removed by a nuclease complex Rad1-Rad10 in budding yeast (XPF-ERCC1 in mammals and Rad16-Swi10 in the fission yeast *Schizosaccharomyces pombe*) [6].

The function of Rad1-Rad10 in SSA requires two positive regulators, Saw1 and Slx4 [7]–[10]. Saw1 recruits Rad1-Rad10 to the DNA substrate during SSA [8],[11]; however, the exact role of Slx4 in SSA is not clear. Furthermore, it is not known whether the activities of the resection nucleases are regulated during SSA.

Here we show that a novel factor Pxd1 is a key regulator of SSA in fission yeast. It interacts with both the nonhomologous ssDNA cleavage nuclease Rad16^XPF^ and the resection nuclease Dna2, thus influencing different aspects of SSA. Interestingly, Pxd1 regulates these two structure-specific nucleases in opposite ways: it promotes the completion of SSA by activating the nuclease activity of Rad16, while it minimizes genetic information loss by inhibiting RPA-mediated Dna2 activation.

## Results

### Identification of Pxd1 as a Rad16- and Dna2-Interacting Protein

**Figure 1:**
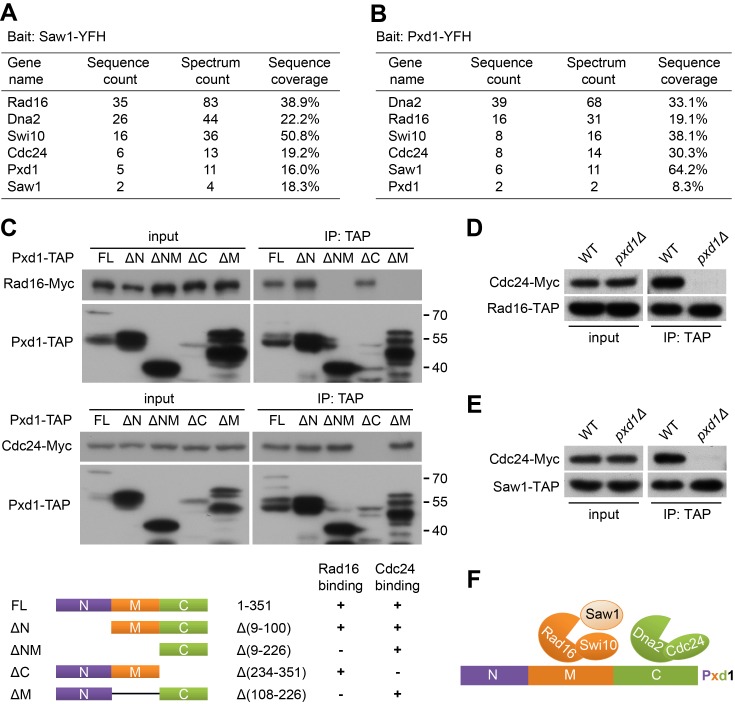
Pxd1 interacts with Rad16-Swi10 and Dna2-Cdc24. (A) Proteins that co-purified with Saw1. Saw1 tagged with a YFP-FLAG-His_6_ (YFH) tag was purified using anti-YFP beads and analyzed by mass spectrometry. (B) Proteins that co-purified with Pxd1. (C) Pxd1 interacts with Rad16-Swi10 and Dna2-Cdc24 through its middle and C-terminal regions, respectively. Full-length (FL) and truncated Pxd1 proteins fused with the TAP tag were immunoprecipitated using IgG beads. The co-immunoprecipitation of Myc-tagged Rad16 or Cdc24 was analyzed by immunoblotting. The N-terminal region of Pxd1 is prone to be cleaved off by proteolysis. The bottom panel depicts the Pxd1 truncations and summarizes the co-immunoprecipitation results. (D) Pxd1 is required for the association between Rad16 and Cdc24. Cdc24-Myc was co-immunoprecipitated with Rad16-TAP in the wild-type background, but not in the *pxd1Δ* background. (E) Pxd1 is required for the association between Saw1 and Cdc24. Cdc24-Myc was co-immunoprecipitated with Saw1-TAP in the wild-type background, but not in the *pxd1Δ* background. (F) Schematic of the inferred organization of the PXD complex.

A previously uncharacterized fission yeast protein, SPBC409.16c, has been predicted by PomBase as the ortholog of budding yeast Saw1 [12]. In budding yeast, Saw1 interacts with the Rad1-Rad10 nuclease [8],[11]. In an affinity purification coupled with mass spectrometry (AP-MS) experiment, we found that Rad16 and Swi10, the fission yeast counterparts of budding yeast Rad1 and Rad10, respectively [13],[14], co-purified with SPBC409.16c (Figure 1A), thus corroborating the PomBase orthology prediction. We will hereafter refer to SPBC409.16c as Saw1.

Intriguingly, Dna2, Cdc24, and an uncharacterized protein SPCC1322.02 also co-purified with Saw1 (Figure 1A). Dna2 and the fission-yeast-unique protein Cdc24 are known to form a heterodimer and are both required for Okazaki fragment maturation in fission yeast [15]. When SPCC1322.02 was used as bait for AP-MS analysis, the same six proteins were again isolated together (Figure 1B), suggesting that Rad16-Swi10-Saw1, Dna2-Cdc24, and SPCC1322.02 co-exist in a protein complex, which we named the PXD (*pombe* XPF and Dna2) complex. Accordingly, we named SPCC1322.02 Pxd1.

### Pxd1 Mediates the Association between Rad16-Swi10-Saw1 and Dna2-Cdc24

Pxd1 is annotated by PomBase as a “sequence orphan” with no apparent orthologs outside of the fission yeast clade, and it does not contain any known domains. To identify the regions of Pxd1 that participate in its interactions with Rad16-Swi10 and Dna2-Cdc24, we performed truncation analysis and found that its interaction with Rad16-Swi10 is mediated by the middle region of Pxd1 (residues 101–233), whereas its interaction with Dna2-Cdc24 is mediated by the C-terminal region of Pxd1 (residues 227–351) (Figure 1C).

Because distinct regions of Pxd1 mediate its interactions with Rad16-Swi10 and Dna2-Cdc24, we hypothesized that Pxd1 may act as a scaffold to bring these two nucleases together. We tested this idea by examining the association of the two nucleases in wild-type and *pxd1Δ* backgrounds. Cdc24 co-immunoprecipitated with Rad16 in the wild type, but this interaction was abolished in *pxd1Δ* (Figure 1D). Similarly, the interaction between Saw1 and Cdc24 was abolished in *pxd1Δ* (Figure 1E). These results suggest that, within the PXD complex, Pxd1 acts as a physical link between the Rad16-Swi10-Saw1 and Dna2-Cdc24 subcomplexes (Figure 1F).

To determine where Pxd1 binds on its binding partners, we performed yeast two-hybrid (Y2H) assay, immunoprecipitation using truncated proteins, and cross-linking mass spectrometry (CXMS) (Figure S1). Rad16, Dna2 and Cdc24, but not Swi10, exhibited positive Y2H interactions with Pxd1. An N-terminal fragment of Rad16 (residues 1–451), which contains a helicase-like domain, was sufficient to co-immunoprecipitate Pxd1 in the absence of Swi10. CXMS analysis of a Dna2-Cdc24-Pxd1(227–351) complex detected cross-links between the K148 residue of Cdc24 and two different residues of Pxd1 (K276 and K351). Consistently, Cdc24(80–245), which contains the K148 residue, is the smallest fragment of Cdc24 that could robustly co-immunoprecipitate Pxd1.

### Pxd1 Acts with Rad16-Swi10 in the IR Response

**Figure 2:**
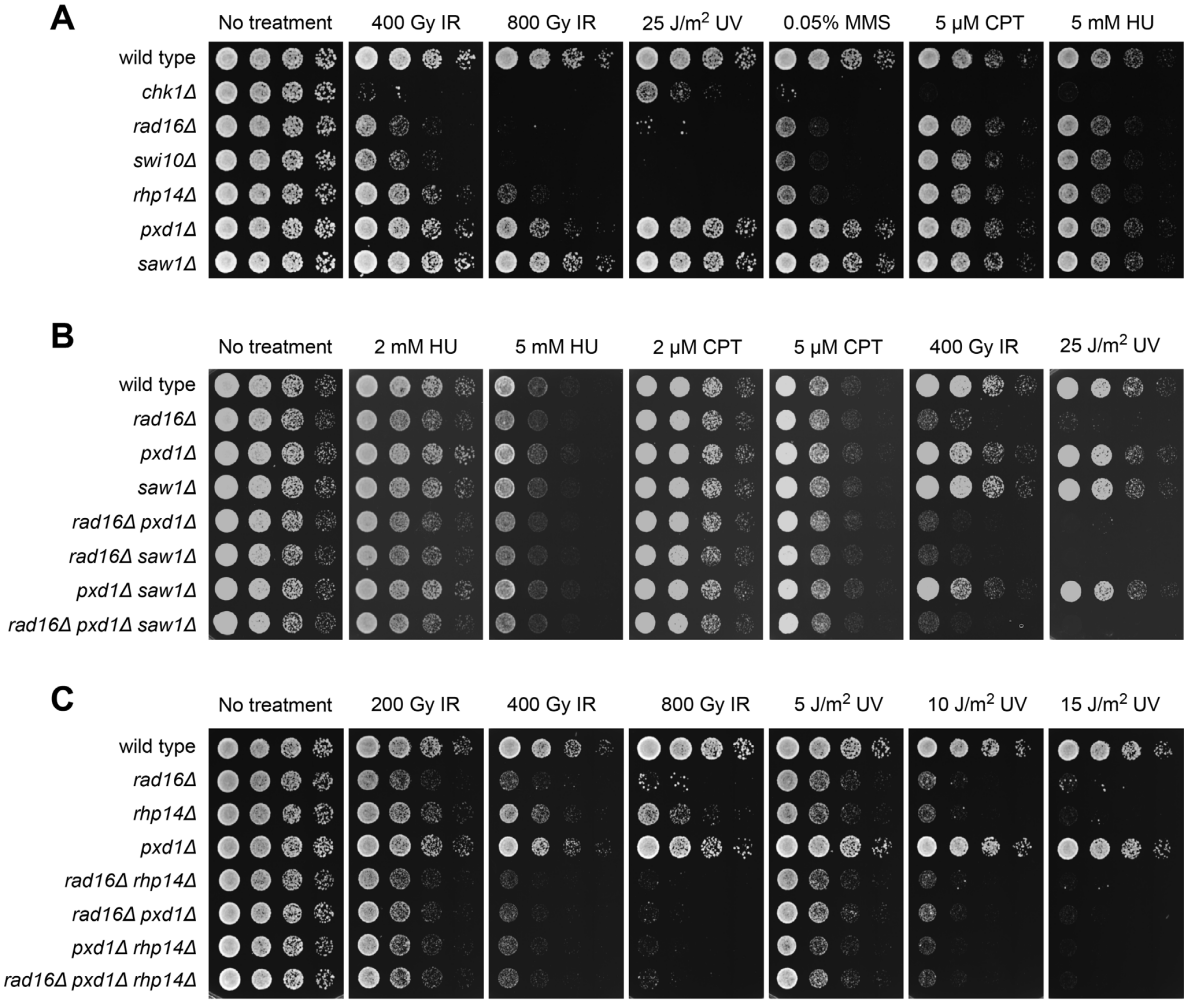
Pxd1 acts with Rad16-Swi10 in the IR response. (A) The DNA damage sensitivity of the indicated strains was examined using a spot assay. *pxd1Δ*, but not *saw1Δ*, cells exhibited mild IR sensitivity. The IR sensitivity of *rad16Δ* and *swi10Δ* cells was stronger than that of NER-defective *rhp14Δ* cells, suggesting a role of Rad16-Swi10 in non-NER repair. (B) Deletion of *saw1* did not alter the DNA damage sensitivity of *pxd1Δ*, *rad16Δ*, or their double mutant. (C) For the IR sensitivity phenotype, *rad16Δ* is epistatic to *rhp14Δ* and *pxd1Δ*. The double mutant *rhp14Δ pxd1Δ* was more sensitive than *rhp14Δ* or *pxd1Δ* and phenocopied *rad16Δ*, suggesting that Pxd1 acts with Rad16 in the non-NER repair process.

To understand the function of Pxd1, we generated a *pxd1* deletion mutant, which exhibited no growth defect (Figure 2A). Thus, Pxd1 is unlikely to be important for the replication function of Dna2-Cdc24, which is essential for viability. We then examined the DNA damage sensitivity of deletion mutants of *pxd1* and related nonessential genes. *pxd1Δ* showed mild sensitivity to ionizing radiation (IR) but displayed no obvious sensitivity to UV, methyl methanesulfonate (MMS), camptothecin (CPT), or hydroxyurea (HU) (Figure 2A). Consistent with the known role of Rad16-Swi10 in nucleotide excision repair (NER), *rad16Δ* and *swi10Δ* showed severe sensitivity to UV that was at a level similar to the mutant lacking another NER factor, Rhp14^XPA^ (Figure 2A). These three mutants also showed similar sensitivity to MMS and HU. However, *rad16Δ* and *swi10Δ* were more sensitive to IR than *rhp14Δ*, which most likely reflected the non-NER functions of Rad16-Swi10, such as the removal of the 3′ nonhomologous ssDNA tails during homologous recombination (HR) repair [16],[17]. Surprisingly, *saw1Δ* displayed no sensitivity to any treatment (Figure 2A). In addition, deletion of *saw1* did not enhance the DNA damage sensitivity of *pxd1Δ* (Figure 2B).

To test the epistatic relationship between *pxd1Δ*, *rhp14Δ*, and *rad16Δ*, we examined the sensitivity of their single, double, and triple mutants (Figure 2C). Deletion of *pxd1*, *rhp14*, or both in *rad16Δ* did not enhance the IR sensitivity. In contrast, the *pxd1Δ rhp14Δ* double mutant showed greater IR sensitivity than either single mutant, reaching a level similar to that of *rad16Δ*. These results suggest that Pxd1 acts with Rad16-Swi10 in the non-NER repair of IR-induced DNA damage.

### Pxd1 Acts with Rad16-Swi10 in SSA

**Figure 3:**
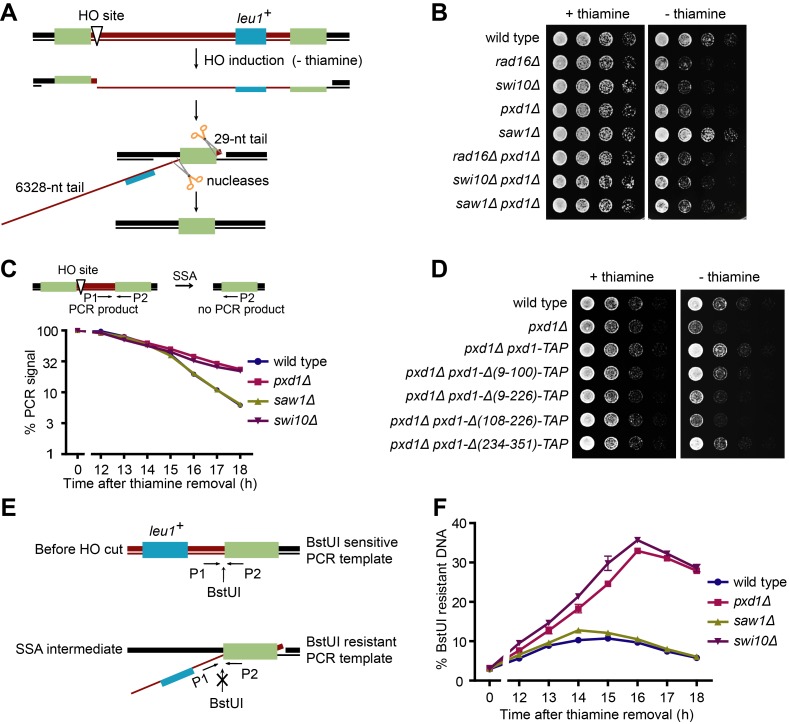
Pxd1 is required for SSA and acts with Rad16-Swi10 in the removal of the 3′ nonhomologous ssDNA tails. (A) Schematic of the SSA system. An HO site is flanked by two 1.2-kb-long direct repeats, shown as green rectangles. HO is controlled by the thiamine-repressible *nmt1* promoter. Upon DSB formation at the HO site, SSA proceeds through resection, annealing between the repeat DNA, cleavage of the 3′ nonhomologous ssDNA tails, gap filling, and ligation. To visually distinguish the two strands of the duplex DNA, they are shown as lines of different thickness. (B) The growth of *rad16Δ*, *swi10Δ*, and *pxd1Δ*, but not *saw1Δ*, cells was delayed by the induction of an SSA-repairable DSB. Serial dilutions of strains harboring the SSA system, shown in (A), were spotted on plates with or without thiamine after incubation in thiamine-free liquid medium for 8 h. (C) *swi10Δ* and *pxd1Δ*, but not *saw1Δ*, cells are defective in the elimination of the intervening sequence between the repeats. The top panel depicts the primers used for qPCR. It takes more than 10 h for HO to be induced after thiamine removal. Data shown are the mean and standard error of assays run in triplicate and are representative of three independent experiments. For most of the data points, error bars are shorter than the size of symbols. (D) The middle region of Pxd1 is required for its SSA function. SSA assay was performed as in (B). (E) Schematic of the assay monitoring the removal of a 3′ nonhomologous ssDNA tail. Prior to qPCR, the genomic DNA was treated with BstUI, which cuts duplex DNA but not ssDNA. (F) *pxd1Δ* and *swi10Δ*, but not *saw1Δ*, cells are defective in 3′ nonhomologous ssDNA removal. The assay depicted in (E) was used. Data shown are the mean and standard error of assays run in triplicate and are representative of three independent experiments. For most of the data points, error bars are shorter than the size of symbols.

To further delineate the role of Pxd1 in non-NER repair, we examined whether Pxd1 functions with Rad16-Swi10 in SSA. We constructed a strain in which an HO endonuclease-induced DSB is flanked by two direct repeats (Figure 3A). In such a system, homologous recombination between the two repeats may proceed through either the SSA or BIR mechanisms, but because the two repeats are only about 6 kb apart, SSA is expected to be the predominant pathway [18]. Regardless of which mechanism is used, two 3′ nonhomologous ssDNA tails, one 6,328 nt long and the other 29 nt long, must be removed by a nuclease such as Rad16-Swi10, resulting in the loss of the HO cleavage site and a *leu1^+^* marker (Figure 3A). For simplicity, we will hereafter refer to this repair process as SSA.

When wild-type cells harboring the SSA system were shifted from an HO repression (+ thiamine) to an HO induction condition (−thiamine) in liquid media, no obvious growth arrest was observed, but the cells became Leu^−^ (Figure S2A and B), indicating that SSA repair was highly efficient. In contrast, when HO was induced in *rad16Δ* and *swi10Δ* cells, their proliferation was retarded for approximately 20 h, suggesting a delay of the repair process (Figure S2A). Eventually most of the *rad16Δ* and *swi10Δ* cells survived and became Leu^−^, most likely due to backup nuclease activities (Figure S2B). On thiamine-free solid media, the repair defect of *rad16Δ* and *swi10Δ* also manifested as a growth delay (Figure 3B). *pxd1Δ* cells showed the same growth delay as *rad16Δ* and *swi10Δ* cells (Figure 3B). In addition, the double mutants *rad16Δ pxd1Δ* and *swi10Δ pxd1Δ* exhibited the same phenotype as the three single mutants, indicating that Rad16-Swi10 and Pxd1 function in the same process. In this assay, *saw1Δ* again behaved like the wild type. Moreover, deleting *saw1* in *pxd1Δ* did not exacerbate the phenotype. Thus, unlike its budding yeast ortholog, fission yeast Saw1 does not appear to be important for SSA.

To more directly monitor SSA, we examined the elimination of the intervening DNA sequence between the repeats using qPCR (Figure 3C). The rate of DNA elimination in the *pxd1Δ* and *swi10Δ* mutants was significantly slower than in the wild type and the *saw1Δ* mutant (Figure 3C). In addition, we visualized Rad52 nuclear foci, which is an indication of ongoing DNA repair activity. In the wild-type and *saw1Δ* cells, the level of Rad52 foci transiently increased after HO induction but returned to the pre-induction level within 8 h (Figure S2C and D). In contrast, in *pxd1Δ*, *rad16Δ*, and *swi10Δ* cells, HO-induced Rad52 foci remained at a high level for more than 10 h. Thus, DNA repair in these three mutants failed to efficiently proceed to completion.

To test whether the interaction between Pxd1 and Rad16-Swi10 is required for SSA, we examined cells expressing truncated versions of Pxd1. Pxd1 missing either its N-terminal region or C-terminal region could rescue the defect of *pxd1Δ*, whereas Pxd1 without the middle region failed to rescue the phenotype (Figure 3D). Thus, the region of Pxd1 involved in Rad16-Swi10 binding is required for SSA.

### Pxd1 Is Required for 3′ Nonhomologous ssDNA Removal in SSA

During SSA, the role of Rad16-Swi10 is to remove the 3′ nonhomologous ssDNA tails. Given that the interaction between Pxd1 and Rad16-Swi10 is required for SSA, we hypothesized that Pxd1 is involved in the same step. To test this idea, we monitored the level of 3′ ssDNA using a qPCR assay. In this assay, the PCR template was genomic DNA pre-digested with a restriction enzyme, BstUI, that cuts double-stranded but not single-stranded DNA. Thus, the level of the PCR product reflects the amount of ssDNA (Figure 3E). In wild-type and *saw1Δ* cells, only a transient and small increase (approximately 10%) of ssDNA occurred after HO induction (Figure 3F). In contrast, in *pxd1Δ* and *swi10Δ* cells, ssDNA accumulated to a much higher level and persisted (Figure 3F). Thus, 3′ ssDNA removal is defective in *pxd1Δ* and *swi10Δ*, but not in *saw1Δ*, mutants.

### Pxd1 Acts with Rad16-Swi10 in Mating-Type Switching and the Removal of Top1 Cleavage Complexes (Top1cc)

**Figure 4:**
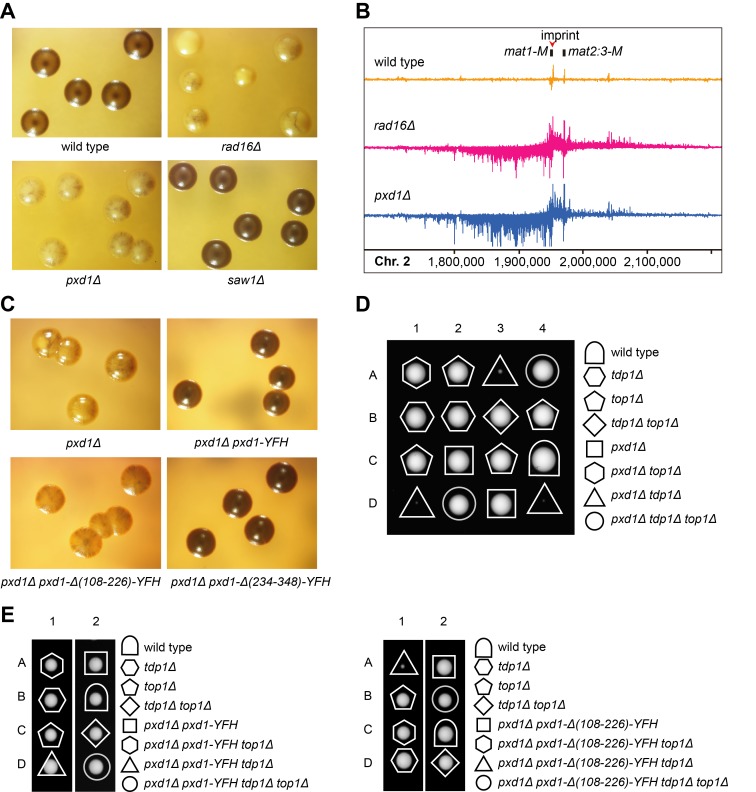
Pxd1 acts with Rad16-Swi10 in mating-type switching and the removal of covalent Top1-DNA adducts. (A) *rad16Δ* and *pxd1Δ*, but not *saw1Δ*, cells are defective in mating-type switching. The *h^90^* strains with the indicated genotypes were spread onto malt extract (ME) plates, and single colonies were allowed to form before they were stained with iodine vapor. (B) Increased Rad52 accumulation at the mating type locus was observed in *rad16Δ h^−^* and *pxd1Δ h^−^* cells. Strand-specific Rad52 ChIP-seq was performed as described [43]. (C) The middle region of Pxd1 is required for its mating-type switching function. Mating-type switching assay was performed as in (A). (D) *tdp1Δ* and *pxd1Δ* are synthetic lethal/sick in a Top1-dependent manner. Representative tetrads from a cross between a *pxd1Δ* strain and a *top1Δ tdp1Δ* strain are shown. (E) The middle region of Pxd1 is required to rescue the synthetic lethality/sickness of the *tdp1Δ pxd1Δ* cells. Shown are representative tetrads from crosses between *pxd1Δ* strains transformed with a plasmid expressing full-length or middle-region-deleted Pxd1 and a *top1Δ tdp1Δ* strain. The plasmid was integrated at the *pxd1* locus.

Rad16 (also known as Swi9) and Swi10 are required for mating-type switching, presumably due to their involvement in resolving recombination intermediates of the HR process triggered by the programmed DSB at the mating type locus (Figure S3A) [19],[20]. To test whether Pxd1 also participates in mating-type switching, we performed an iodine-staining assay on *h^90^* homothallic strains growing on a medium compatible with mating and sporulation (Figure S3B). Dark staining indicates efficient mating-type switching, whereas light or sectored staining indicates defects in mating-type switching. Wild-type and *saw1Δ h^90^* colonies were darkly and homogenously stained (Figure 4A). In contrast, *rad16Δ* and *pxd1Δ* colonies showed much weaker and uneven staining patterns. This result suggests that *pxd1Δ*, like *rad16Δ*, is defective in mating-type switching. Consistent with the idea that a failure of the HR process underlies the mating-type switching defect of *rad16Δ* and *pxd1Δ*, we observed using ChIP-seq that, in heterothallic *h^−^* cells, Rad52 accumulated more strongly at the mating type locus in *rad16Δ* and *pxd1Δ* than in wild-type cells (Figure 4B). In *h^−^* cells, the programmed DSB also triggers an HR process, but the mating type does not switch because only one type of donor sequence is available.

When different truncated forms of Pxd1 were tested for their abilities to rescue the mating-type switching defect, the middle region-deleted version of Pxd1 failed to rescue the iodine-staining phenotype of *pxd1Δ h^90^* colonies, suggesting that the interaction between Pxd1 and Rad16-Swi10 is important for mating-type switching (Figure 4C).

Covalent Top1-DNA adducts, referred to as Top1 cleavage complexes (Top1cc), arise spontaneously and can jeopardize cell survival if not removed. It was shown recently that Rad16-Swi10 and Tdp1 redundantly remove Top1cc in fission yeast [21]. We, therefore, tested whether Pxd1 also contributes to this process. Tetrad analysis showed that, like *swi10Δ*, *pxd1Δ* is synthetic lethal/sick with *tdp1Δ*, and the synthetic lethality/sickness can be rescued by the deletion of *top1* (Figure 4D and Figure S4A). Further analysis showed that the C-terminally truncated version, but not the middle region-deleted version, of Pxd1 could rescue the synthetic lethality/sickness (Figure 4E and Figure S4B). These results suggest that Pxd1 acts with Rad16-Swi10 in the removal of Top1cc (Figure S4C).

### Pxd1 Activates the 3′ Endonuclease Activity of Rad16-Swi10

**Figure 5:**
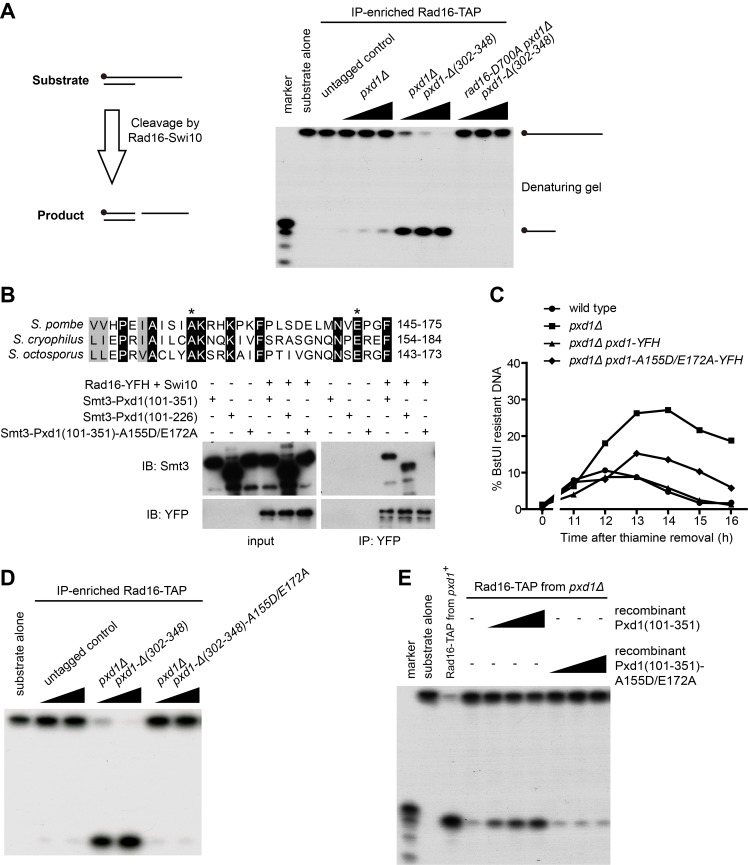
Pxd1 can activate the 3′ DNA endonuclease activity of Rad16-Swi10. (A) Pxd1 is required for robust 3′ nuclease activity of Rad16-Swi10. The nuclease activity of Rad16-TAP expressed at the endogenous level and immunoprecipitated from the indicated strains was examined using a 3′ overhang DNA substrate. Schematics of the substrate and the product are shown at the left side of the panel. The 5′ ^32^P radiolabel is indicated by a black dot. The reaction mixtures were subjected to 15% denaturing PAGE and autoradiography. The wedge symbols represent increasing (two-fold) amounts of Rad16-TAP. (B) The A155D/E172A mutation in the middle region of Pxd1 diminished the interaction between Pxd1 and Rad16-Swi10. A155 and E172 are conserved in the Pxd1 homologs from two other *Schizosaccharomyces* species and are labeled by the asterisks in the sequence alignment. Rad16-YFH and Swi10 purified from fission yeast were incubated with Smt3-tagged Pxd1 fragments purified from *E. coli*. Anti-YFP beads were used to retrieve the Rad16-Swi10 complex, and the co-immunoprecipitation of Pxd1 was analyzed using immunoblotting. (C) The A155D/E172A mutation hampered the ability of Pxd1 to support the removal of the 3′ nonhomologous ssDNA during SSA. The assay was performed as in Figure 3F. (D) The A155D/E172A mutation disrupted the ability of Pxd1 to enhance the nuclease activity of Rad16-Swi10. The assay was performed as in (A). The wedge symbols represent increasing (two-fold) amounts of Rad16-TAP. (E) Recombinant Pxd1 can activate the nuclease activity of Rad16-Swi10. Rad16-TAP from a *pxd1Δ pxd1-Δ (302–348)* strain (denoted as *pxd1^+^*) was used as a positive control. The nuclease activity of Rad16-TAP from a *pxd1Δ* strain was determined after incubating with different amounts of Pxd1(101–351) or Pxd1(101–351)-A155D/E172A purified from *E. coli*. The assay was performed as in (A). The wedge symbols represent 0.5, 1, and 5 µg of recombinant Pxd1 protein.

To understand how Pxd1 acts with Rad16-Swi10, we tested whether its absence affects the nuclease activity of Rad16-Swi10 purified from fission yeast cells. For a positive control, we used a strain expressing C-terminally truncated Pxd1 as the only form of Pxd1, so that Dna2-Cdc24, which also has nuclease activities, does not co-purify with Rad16-Swi10. As described earlier, this truncated form of Pxd1 is sufficient for SSA, mating-type switching, and Top1cc removal. Consistent with the known substrate specificity of XPF family nucleases, Rad16 immunoprecipitated from such a strain showed robust nuclease activity toward 3′ overhang DNA and Y fork DNA but not 5′ overhang DNA (Figure S5A). The nuclease-dead mutant Rad16-D700A immunoprecipitated from the same Pxd1 C-terminal truncation background did not show nuclease activity toward any substrates, demonstrating that the nuclease activity we observed was Rad16-specific (Figure S5A). Rad16 immunoprecipitated from *pxd1Δ* cells had much weaker nuclease activity than the positive control (Figure 5A and Figure S5B). The expression level and stability of Rad16 were not affected by the loss of Pxd1 (Figure S5C). Thus, Pxd1 is required for a robust nuclease activity of Rad16-Swi10.

The middle region of Pxd1 is required for its interaction with Rad16-Swi10 and is needed for SSA, mating-type switching, and the removal of Top1cc. To identify functionally important residues within this region, we mutated the residues conserved between Pxd1 and its homologs in two other fission yeast species and found that a double point mutation, A155D/E172A, significantly weakened the interaction between a recombinant Pxd1 protein purified from *E. coli* and Rad16-Swi10 immunoprecipitated from *pxd1Δ* fission yeast cells (Figure 5B). When introduced into the *pxd1* gene in fission yeast, this mutation impaired 3′ ssDNA removal during SSA (Figure 5C) and diminished the nuclease activity of Rad16-Swi10 purified from the Pxd1 C-terminal truncation background (Figure 5D). These data strongly suggest that the interaction between Pxd1 and Rad16-Swi10 is needed for Pxd1 to activate Rad16-Swi10.

When we added Pxd1 protein purified from *E. coli* to Rad16-Swi10 immunoprecipitated from *pxd1Δ* cells, we observed a dose-dependent enhancement of nuclease activity (Figure 5E). As a control, the A155D/E172A mutant form of Pxd1 purified from *E. coli* failed to activate the nuclease activity (Figure 5E). Thus, recombinant Pxd1 is sufficient for activating Rad16-Swi10.

### Overexpression of a Pxd1 C-Terminal Fragment Inhibits the Function of Dna2-Cdc24

**Figure 6:**
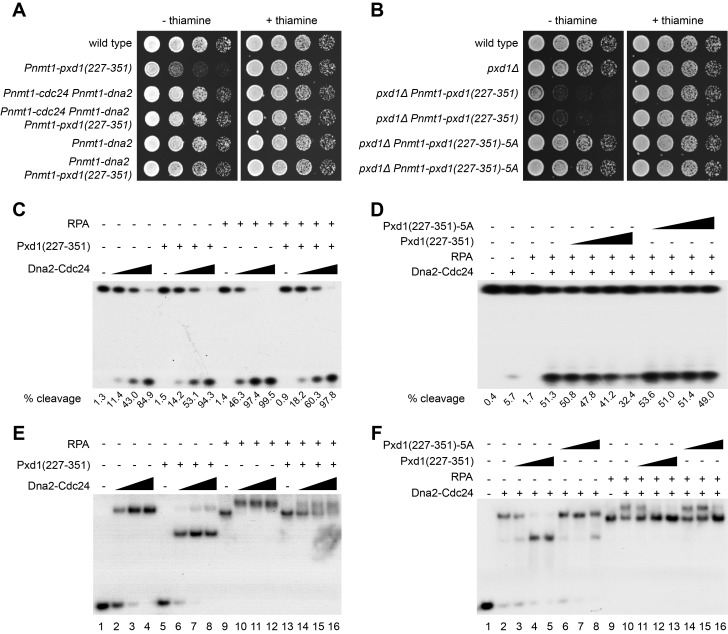
Pxd1 prevents RPA-mediated activation of Dna2 nuclease activity. (A) Overexpression of a Pxd1 C-terminal fragment, Pxd1(227–351), caused growth inhibition and this phenotype can be suppressed by co-overexpression of Dna2 and Cdc24 or overexpression of Dna2 alone. (B) The 5A mutation that weakens the interaction between Pxd1 and Dna2-Cdc24 abolished the ability of Pxd1(227–351) to inhibit growth. (C) Pxd1(227–351) purified from *E. coli* inhibited the stimulation of the 5′ nuclease activity of Dna2-Cdc24 by RPA, but did not affect the basal nuclease activity of Dna2-Cdc24. Dna2 and Cdc24-YFH were co-overexpressed in *pxd1Δ* cells and purified using anti-FLAG beads. Recombinant RPA was purified from *E. coli*. The nuclease reaction products were analyzed using 15% denaturing PAGE gels and autoradiography. The percentage of cleavage (% cleavage) was determined as the percentage of the substrate that is converted to the product. The amounts of proteins used were as follows: Dna2-Cdc24, 50, 125, or 250 ng; RPA, 25 ng; Pxd1, 250 ng. (D) The 5A mutation weakened the ability of Pxd1 to inhibit the RPA-stimulated nuclease activity of Dna2-Cdc24. The assay was performed as in (C). The amounts of proteins used were as follows: Dna2-Cdc24, 125 ng; RPA, 125 ng; Pxd1, 15, 30, 62.5, or 125 ng. (E) Pxd1 inhibited the association of Dna2-Cdc24 with an RPA-DNA complex in a gel shift analysis. Increasing amounts of Dna2-Cdc24 were incubated with a 5′ overhang DNA in the presence (+) or absence (−) of Pxd1(227–351) and RPA. The mixture was analyzed using 5% native PAGE gel and autoradiography. The amounts of proteins used were as follows: Dna2-Cdc24, 100, 150, or 300 ng; RPA, 50 ng; Pxd1, 500 ng. (F) The 5A mutation weakened the ability of Pxd1 to inhibit the association of Dna2-Cdc24 with an RPA-DNA complex. Dna2, RPA, and increasing amounts of Pxd1(227–351) or Pxd1(227–351)-5A were used for gel shift analysis. The mixture was analyzed as in (E). The amounts of proteins used were as follows: Dna2-Cdc24, 200 ng; RPA, 50 ng; Pxd1, 10, 100, or 500 ng.

To probe the role of the interaction between Pxd1 and Dna2-Cdc24, we overexpressed a Pxd1 C-terminal fragment, Pxd1(227–351), which encompasses the Dna2-Cdc24–interacting region. Remarkably, Pxd1(227–351) overexpression caused severe growth defect, and this defect could be suppressed by co-overexpression of both Dna2 and Cdc24, or Dna2 alone (Figure 6A). Two mutant alleles of the gene encoding the DNA helicase Pfh1 (Pif1 homolog), *pfh1-R20* and *pfh1-R23*, which are suppressors of temperature-sensitive mutants of *dna2* and *cdc24* [22],[23], also suppressed the growth defect caused by Pxd1(227–351) overexpression (Figure S6A). Thus, the growth defect is likely due to a down-regulation of the functions of Dna2-Cdc24. To determine whether the interaction between Pxd1 and Dna2-Cdc24 is important for this down-regulation, we performed mutagenesis on the C-terminal region of Pxd1 and found that simultaneously mutating five residues conserved between Pxd1 and its homologs in two other fission yeast species, referred to as the 5A mutation, weakened the interaction between Pxd1 and Dna2-Cdc24 (Figure S6B). The overexpression of Pxd1(227–351)-5A did not cause any growth defect (Figure 6B), indicating that the Pxd1(227–351) overexpression phenotype is mediated by an interaction with Dna2-Cdc24.

### Pxd1 Blocks the RPA-Mediated Activation of the Nuclease Activity of Dna2

To understand how Pxd1(227–351) down-regulates the functions of Dna2-Cdc24 when overexpressed, we investigated whether in vitro it influences the nuclease activity of Dna2-Cdc24. We found that Dna2 and Cdc24 co-overexpressed and purified from *pxd1Δ* cells were able to cleave a 5′ overhang DNA substrate (Figure 6C). The stability of Dna2 and Cdc24 was not affected by *pxd1Δ* (Figure S6C). Consistent with the results obtained with budding yeast and human Dna2 [24],[25], the addition of RPA markedly stimulated the nuclease activity of Dna2. Recombinant Pxd1(227–351) purified from *E. coli* did not affect the basal activity of Dna2; however, it significantly weakened the activation effect of RPA (Figure 6C). Pxd1(227–351)-5A failed to inhibit the RPA-mediated activation of Dna2 (Figure 6D). Thus, the interaction between Pxd1 and Dna2 impedes the activation of Dna2 by RPA.

RPA can enhance the nuclease activity of Dna2 by promoting the binding of Dna2 on ssDNA in budding yeast [24]; therefore, we hypothesized that Pxd1(227–351) may block RPA-mediated Dna2 binding to DNA substrates. To test this idea, we first investigated the ability of Pxd1 and Dna2-Cdc24 to bind a 5′ overhang DNA using a gel mobility shift assay. In this assay, DNA cleavage was prevented by using a buffer containing 1 mM EDTA and no divalent cations. Dna2-Cdc24 shifted the mobility of the DNA, whereas Pxd1(227–351) had no effect (Figure 6E, lanes 2–5). The addition of Pxd1(227–351) with Dna2-Cdc24 led to the formation of a complex that migrated faster than the Dna2-Cdc24-DNA complex (Figure 6E, lanes 6–8 and Figure 6F, lanes 3–5), most likely due to a higher negative charge of the Pxd1-Dna2-Cdc24-DNA complex because the recombinant Pxd1(227–351) has a low PI of 5.09. As a control, the addition of Pxd1(227–351)-5A, which cannot efficiently interact with Dna2-Cdc24, had much weaker ability to shift the Dna2-Cdc24-DNA complex (Figure 6F, lanes 6–8). These results show that, consistent with the lack of effect of Pxd1 on the basal nuclease activity of Dna2, Pxd1 does not appear to affect the ability of Dna2-Cdc24 to bind naked DNA.

When RPA was added to the DNA binding reaction with Dna2-Cdc24, a Dna2-Cdc24-RPA-DNA complex that migrated slower than the Dna2-Cdc24-DNA complex and the RPA-DNA complex was detected (Figure 6E, lanes 10–12). Addition of Pxd1(227–351) interfered with the formation of this higher-order complex and resulted in a form of DNA that appeared to be bound by only RPA (Figure 6E, lanes 14–16 and Figure 6F, lanes 11–13), suggesting that Dna2-Cdc24 was dissociated from the RPA-DNA complex in the presence of Pxd1. In comparison, Pxd1(227–351)-5A was weaker in its ability to disrupt the higher-order complex (Figure 6F, lanes 14–16). From these results, we conclude that Pxd1 inhibits the RPA-mediated activation of Dna2 by blocking the binding of Dna2-Cdc24 to RPA-coated DNA.

### Pxd1 Attenuates DNA Resection by Inhibiting the Rqh1-Dna2 Pathway

**Figure 7:**
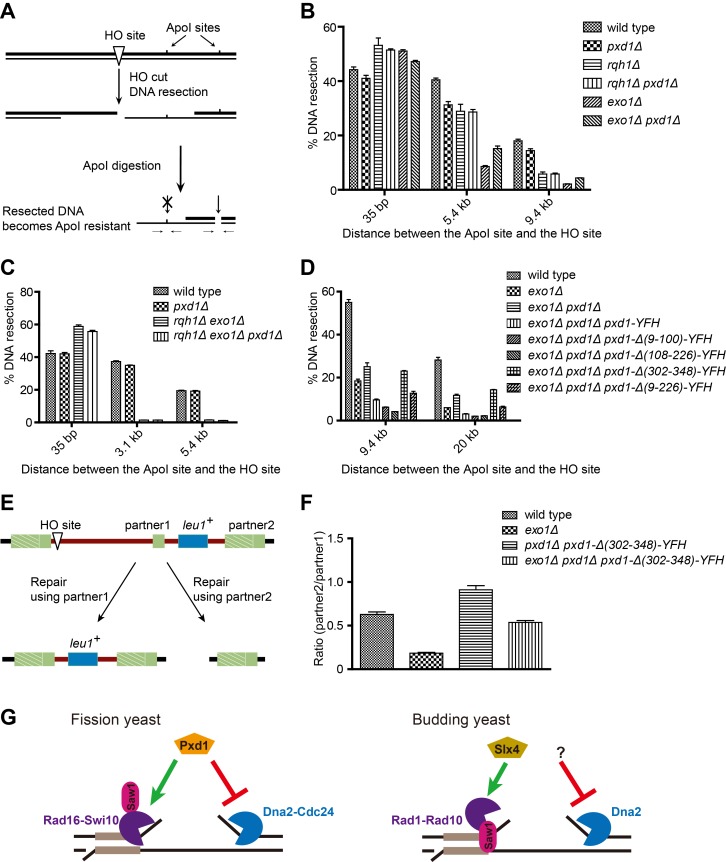
Pxd1 attenuates long-range resection mediated by the Rqh1-Dna2 pathway and restricts the use of a break-distal homologous sequence in SSA repair. (A) Schematic of the resection assay. Resection-generated ssDNA is resistant to ApoI digestion and can be quantitated using qPCR [26]. (B) *pxd1Δ* partially suppressed the resection defect of *exo1Δ*. The levels of DNA resection at different ApoI sites 14 h after thiamine removal were quantitated. See Table S4 for the data that were used to generate the histogram. (C) *pxd1Δ* did not suppress the resection defect of *exo1Δ rqh1Δ*. The levels of DNA resection at different ApoI sites 12 h after thiamine removal were quantitated. See Table S4 for the data that were used to generate the histogram. (D) The C-terminal region of Pxd1 is necessary and sufficient for resection attenuation in an *exo1Δ pxd1Δ* background. The levels of DNA resection at different ApoI sites 14 h after thiamine removal were quantitated. See Table S4 for the data that were used to generate the histogram. (E) Schematic of the SSA competition system. Cells remain Leu^+^ if the DSB-proximal homologous sequence, partner1, is used for SSA repair, but become Leu^−^ if the DSB-distal homologous sequence, partner2, is used for SSA repair. (F) Removing the C-terminal region of Pxd1 increased the usage of partner2 in both *exo1Δ* and *exo1^+^* backgrounds. See Table S4 for the data that were used to generate the histogram. (G) Schematic depicting the roles of Pxd1 and its interactors in SSA repair. For comparison, their equivalents in budding yeast are shown on the right. Unlike its budding yeast ortholog, fission yeast Saw1 is dispensable for SSA, and may not be involved in the targeting of Rad16-Swi10 to the DNA substrate. Budding yeast Slx4 is a positive regulator of Rad1–Rad10, but it is unclear whether it can activate the nuclease activity of Rad1–Rad10.

The Dna2-inhibitory effect of Pxd1 may influence the actions of Dna2 in either DNA replication or DSB resection. Because Pxd1 is down-regulated during the S phase of the cell cycle (our unpublished observation), we hypothesized that it may mainly regulate the resection function of Dna2. During resection, Dna2 is expected to act with Rqh1, a RecQ family helicase, in a pathway parallel to Exo1 [5]; therefore, in an *exo1Δ* background, the residual resection activity should be Rqh1- and Dna2-dependent. Using a qPCR-based assay to monitor resection from an irreparable HO-induced DSB (Figure 7A), we found that, as reported [26], the deletion of *exo1*, but not *rqh1*, strongly reduced long-range resection (Figure 7B). No obvious difference was found between *pxd1Δ* and the wild type. However, deletion of *pxd1* in *exo1Δ* partially rescued the resection defect. Thus, consistent with the results of Pxd1(227–351) overexpression and the in vitro nuclease assay, Pxd1 appears to attenuate the Dna2- and Rqh1-mediated resection activity, at least in the *exo1Δ* background. Supporting this idea, the deletion of *pxd1* did not rescue the DNA resection defect of *rqh1Δ exo1Δ* cells (Figure 7C). The DNA damage sensitivity of *exo1Δ* cells was not rescued by *pxd1Δ* (Figure S6D), probably due to Exo1 also playing nonresection roles in genome maintenance.

To determine which region of Pxd1 is involved in resection inhibition, we examined the effect of introducing truncated versions of Pxd1 into an *exo1Δ pxd1Δ* double mutant. The N-terminal–truncated and middle-region–deleted versions curtailed long-range DNA resection as strongly as the full-length Pxd1. In contrast, a C-terminally truncated version, Pxd1-*Δ* (302–348), which is defective in binding Dna2, failed to impede resection (Figure 7D). These results suggest that the interaction between Pxd1 and Dna2 is required for the inhibitory effect of Pxd1 on DNA resection. In addition, the C-terminal region of Pxd1 alone can inhibit DNA resection in the *exo1Δ pxd1Δ* background (Figure 7D).

### Pxd1 Promotes the Use of a DSB-Proximal Homologous Sequence in SSA Repair

During the SSA repair process, DNA resection is required for rendering the homologous repeats single-stranded [4],[18]. We hypothesized that the C-terminal region of Pxd1 may regulate the homologous partner choice during SSA repair when there are multiple homologous sequences on the same side of the DSB [4],[27]. To test this idea, we constructed an SSA competition system. In this system, one additional homologous sequence was inserted between the two repeats in the original SSA strain (Figure 7E). During SSA repair, the repeat sequence on the left side of the HO site can anneal with either potential homologous partner on the right side of the HO site. If partner1 is used, the postrepair cells will remain Leu^+^; however, if partner2 is used, cells will become Leu^−^ and suffer a greater loss of genetic information (Figure 7E). We found that the DSB-proximal homologous sequence, partner1, was more frequently used in *exo1Δ* than in wild-type cells (Figure 7F), presumably because slower resection in *exo1Δ* cells reduces the chance of partner2 becoming single-stranded before a productive repair using partner1 has occurred. Removing the Dna2-inhibitory region of Pxd1 reversed the effect caused by *exo1* deletion (Figure 7F), consistent with the rescue of the resection defect observed using the irreparable HO system. Interestingly, in an *exo1^+^* background, the same Pxd1 truncation enhanced the use of the distal homologous sequence, partner2 (Figure 7F). These results suggest that Pxd1 restricts the use of break-distal homologous sequences during SSA repair to prevent excessive loss of genetic information.

## Discussion

In this study, we identified a novel fission yeast protein, Pxd1, which interacts with two structure-specific nucleases, Rad16-Swi10 and Dna2-Cdc24. Our data indicate that Pxd1 can activate the 3′ nuclease activity of Rad16-Swi10, but inhibit the RPA-mediated activation of the 5′ nuclease activity of Dna2-Cdc24. These two capacities of Pxd1 allow it to promote SSA and, at the same time, reduce the negative impact of SSA on genome integrity (Figure 7G).

Unlike the situations in budding yeast, in fission yeast, neither *saw1Δ* nor *slx4Δ* has an observable SSA defect (Figure 3 and Figure S5D). Among the two functionally important features of *S. cerevisiae* Saw1 [11], the R19 residue required for Rad1 binding is conserved in *S. pombe* Saw1, whereas the C-terminal positive amino acid stretch required for DNA binding is missing in *S. pombe* Saw1 (Figure S7). We suspect that *S. pombe* Saw1 may have lost its SSA-related function or become redundant.

Compared with Slx4 proteins in *S. cerevisiae* and metazoans, *S. pombe* Slx4 is much shorter and appears to have lost the region required for the interaction with XPF-ERCC1 [28]. On the other hand, the middle region of Pxd1 (residues 101–233), which mediates Rad16 binding, seems to possess sequence similarity to the XPF-binding region of metazoan Slx4 proteins, which has been referred to as the MLR (MEI9^XPF^-interaction-Like Region) (Figure S5E) [28]–[30]. Thus, we speculate that during evolution, in the lineage leading to the fission yeast, the ancestor Slx4 protein may have split into two proteins, one becoming Pxd1 and the other evolving into the current-day *S. pombe* Slx4, which is solely involved in the regulation of the Slx1 nuclease [31].

In budding yeast, CDK1-mediated phosphorylation promotes the resection function of Dna2 [32]. Here we show that the resection activity of fission yeast Dna2 is subject to a negative regulation by Pxd1. Thus, Dna2 appears to be a regulatory target used in diverse organisms for controlling the resection process. Intriguingly, *pxd1* C-terminal truncation caused an overt phenotype in the SSA competition assay, but *pxd1* deletion did not alter resection in the irreparable HO system, suggesting the possibility that the resection process may be regulated differently depending on whether strand annealing with a homologous partner has occurred.

Highly repetitive DNA elements, such as retrotransposons in yeasts and Alu elements in humans, mediate chromosome rearrangements through homologous recombination pathways including SSA [33]–[35]. The results of our SSA competition assay suggest that fine-tuning the resection activities may be a strategy that evolution has exploited to ameliorate the deleterious consequences of repeat-mediated recombination.

Are there evolutionary advantages of using one protein to exert opposite controls on two nucleases? One possibility is that Pxd1 may serve as a hub to integrate regulatory signals so that the up-regulation of one nuclease and the down-regulation of the other can be more precisely coordinated. The expression level of Pxd1 appears to decrease in S phase (our unpublished observation), suggesting that cell cycle control of these two nucleases is imposed through Pxd1. Thus, the activity of Dna2 is relieved from inhibition during S phase when it is needed for DNA replication. On the other hand, given that the activation of Rad16 by Pxd1 is important for removing the 3′ nonhomologous ssDNA, the decrease of Pxd1 during S phase may curtail HR repair events involving nonhomologous ssDNA. Further analysis will be needed to assess to what extent such a regulation affects DNA repair pathway choices.

## Materials and Methods

### Fission Yeast Strains

The fission yeast strains used in this study are listed in Table S1, and plasmids used in this study are listed in Table S2. Genetic methods for strain construction and the composition of media are as described [36]. To construct an SSA system based on a strain in which an HO cleavage site is inserted at the *arg3* locus [37],[38], we first cloned a 1.2-kb sequence immediately upstream of the *arg3* ORF between the EcoRI and ClaI sites in the integrating vector pJK148 [39], resulting in plasmid pDB169. Then, a 0.6-kb sequence corresponding to *cmb1* ORF, which is immediately downstream of *arg3*, was cloned into the BamHI site in pDB169, resulting in plasmid pDB174. A 0.3-kb sequence from the intergenic region between *arg3* and *cmb1* was cloned between the NotI and SacII sites in pDB174, resulting in plasmid pDB176. Integration of XbaI-cut pDB176 into the HO strain DY1012 resulted in the SSA strain DY2392. For monitoring the ssDNA tail removal, a BstUI restriction site was introduced into pDB176, resulting in plasmid pDB459. Integration of pDB459 into the HO strain DY4840 resulted in the SSA strain DY5999. To create the SSA competition system, a 400-bp sequence immediately upstream of the *arg3* ORF was inserted into the AatII site in pDB176, resulting in plasmid pDB1637, which was then integrated into an HO strain. Protein overexpression in *S. pombe* was conducted using pDUAL vectors containing the strong *nmt1* promoter [40],[41].

### Immunoprecipitation

The lysate from 50 OD_600_ units of cells was prepared by glass bead beating in lysis buffer A (50 mM Tris-HCl, pH 8.0, 0.1 M NaCl, 10% glycerol, 0.05% NP-40, 1 mM PMSF, 1 mM DTT, 1× Roche Protease Inhibitor Cocktail). TAP-tagged and YFP-tagged proteins were immunoprecipitated with IgG Sepharose beads (GE healthcare) and GFP-Trap beads (Chromotek), respectively.

### Protein Purification

Rad16-YFH and Swi10 were co-overexpressed in an *isp6Δ psp3Δ pxd1Δ* fission yeast strain. Cells were lysed using a French press in lysis buffer A. YFH-tagged protein was enriched with anti-FLAG M2 affinity gel (Sigma) and eluted with 3× FLAG peptide.

Cdc24-YFH and Dna2 were co-overexpressed and purified as above.

His_6_-tagged RPA and Pxd1 were expressed in a BL21 *E. coli* strain. Cells were lysed using a French press in lysis buffer B (50 mM phosphate buffer, pH 8.0, 0.3 M NaCl, 10 mM imidazole, 10% glycerol, 1 mM PMSF), and purification was performed using Ni-NTA-agarose (QIAGEN). The eluate was dialyzed with storage buffer (50 mM Tris-HCl, pH 8.0, 0.1 M NaCl, 10% glycerol, 1 mM DTT) before freezing at −80°C.

### Yeast Two-Hybrid Analysis

For yeast two-hybrid analysis, we used the Matchmaker system (Clontech). Bait plasmids were constructed by inserting cDNAs into a modified pGBKT7 vector. Prey plasmids were constructed by inserting cDNAs into a modified pGAD GH vector. Bait and prey plasmids were co-transformed into the AH109 strain, and transformants were selected on the double dropout medium (SD/–Leu/–Trp). The activation of the *HIS3* and *ADE2* reporter genes was assessed on the quadruple dropout medium (SD/–Ade/–His/–Leu/–Trp).

### Cross-Linking Mass Spectrometry (CXMS)

Dna2-Cdc24-Pxd1(227–351) complex was prepared by incubating anti-FLAG beads bound by Cdc24-YFH and Dna2 from fission yeast with Pxd1(227–351) from *E. coli*, washing the beads, and eluting with 3× FLAG peptide. About 12 µg of purified complex in a volume of 20 µl was cross-linked by BS3 or DSS at a final concentration of 0.5 mM for 1 h at room temperature. The reactions were quenched with 20 mM NH_4_HCO_3_. Proteins were precipitated with ice-cold acetone, resuspended in 8 M urea, 100 mM Tris, pH 8.5. After trypsin digestion, the LC-MS/MS analysis was performed on an Easy-nLC 1000 UHPLC (Thermo Fisher Scientific) coupled to a Q Exactive-Orbitrap mass spectrometer (Thermo Fisher Scientific). Peptides were loaded on a pre-column (75 µm ID, 8 cm long, packed with ODS-AQ 12 nm–10 µm beads from YMC Co., Ltd.) and separated on an analytical column (75 µm ID, 11 cm long, packed with Luna C18 3 µm 100 Å resin from Phenomenex) using an acetonitrile gradient from 0–25% in 55 min at a flow rate of 200 nl/min. The top 10 most intense precursor ions from each full scan (resolution 70,000) were isolated for HCD MS2 (resolution 17,500; NCE 27) with a dynamic exclusion time of 60 s. Precursors with 1+, 2+, or unassigned charge states were excluded. pLink was used to identified cross-linked peptides with the cutoffs of FDR<5% and E_value<0.001 [42].

### Spot Assay

For MMS, CPT, and HU sensitivity analysis, five-fold serial dilutions of cells were spotted onto YES with or without the indicated concentration of the chemical. To measure UV sensitivity, after spotting on YES plates, the cells were exposed to the indicated dose of UV treatment. To measure IR sensitivity, the cells were irradiated in microfuge tubes using a Cesium-137 Gammacell 1000 irradiator and then spotted onto YES. The plates were incubated for 2 or 3 d at 30°C.

### ssDNA Tail Removal Assay

Genomic DNA was extracted from 3–5 OD_600_ units of cells collected at different times after HO induction. Five hundred nanograms of genome DNA was digested by 4 U of BstUI for 1.5 h. The amount of amplifiable DNA was determined by qPCR, using the actin gene, *act1*, as the normalization control. Primer sequences are listed in Table S3.

### DSB Resection Assay

Genomic DNA was extracted from 3–5 OD_600_ units of cells collected at different times after HO induction. Five hundred nanograms of genome DNA was digested by 4 U of ApoI for 1.5 h. The amount of amplifiable DNA was determined by qPCR. Primers located at different distances from the HO site were used, and their sequences are listed in Table S3.

The following formula was used to calculate the percentage of DNA that was resected: %resected = (100/2^ΔCt−1^)/f. ΔCt is the difference in average cycles between digested template and undigested template, and f is the fraction of DNA that has been cut by HO.

### DNA Substrates of Nuclease Assays

Oligo461 (5′-CACGCTACCGAATTCTGACTTGCTAGGACATCTTTGCCCACGTTGACCC-3′) and oligo462 (5′-GTCAGAATTCGGTAGCGTG-3′) were used to prepare the 3′ overhang DNA structure. Oligo461 and oligo463 (5′-GGGTCAACGTGGGCAAAG-3′) were used to prepare the 5′ overhang DNA structure. Oligo461 and oligo464 (5′-TCGATAGTCTCTAGATAGCATGTCCTAGCAAGTCAGAATTCGGTAGCGTG-3′) were used to prepare the Y fork DNA structure. The oligos were annealed in 1× annealing buffer (50 mM Tris-HCl, pH 7.5, 100 mM NaCl). For radiolabeled substrates, oligo461 was radiolabeled at its 5′ end. For reactions analyzed with ethidium bromide (EB) staining, 30 pmol of nonradioactive substrate was used per reaction. For reactions analyzed with autoradiography, 30 pmol of nonradioactive substrate mixed with about 50 fmol of radioactive substrate was used per reaction.

### Endonuclease Assays for Rad16-Swi10

Anti-TAP immunoprecipitates from 50 OD_600_ units of cells were incubated with substrate in 50 mM Tris-HCl, pH 7.5, 50 mM NaCl, 1 mM MnCl_2_, 1 mM dithiothreitol, and 0.1 mg/ml bovine serum albumin (BSA) at 30°C for 1 h. The products were separated in 15% denaturing or 10% native gels. The substrates used for denaturing gel analysis were radiolabeled, whereas the substrates for native gel analysis were not radiolabeled. The native PAGE gels were stained with EB, and the denaturing PAGE gels were analyzed by autoradiography.

### Endonuclease Assays for Dna2-Cdc24

The reaction mixtures (20 µl) contained 50 mM Tris-HCl, pH 7.5, 50 mM NaCl, 1 mM MgCl_2_, 1 mM dithiothreitol, 0.1 mg/ml BSA, and 30 pmol of substrate. Reactions were carried out at 30°C for 1 h, and the products were analyzed in a 15% denaturing gel.

### Gel Shift Assay

The assay mixtures (10 µl) contained 50 mM Tris-HCl, pH 7.5, 1 mM EDTA, 1 mM dithiothreitol, 0.1 mg/ml BSA, 50 mM NaCl, 5% glycerol, and 15 fmol of radioactive 5′ overhang DNA. The assay mixtures were incubated at room temperature for 30 min, and then 2 µl of 6× native loading buffer was added. The products were separated in a 5% PAGE gel in 1× TBE at 3 W for 2 h and analyzed by autoradiography.

## Supporting Information

Figure S1 **Mapping the binding interfaces on the Pxd1-binding proteins.** (A) In yeast two-hybrid assays, Pxd1 interacts with Rad16 and Dna2 as a bait and interacts with Cdc24 as a prey. The interactions were scored according to the growth on the quadruple dropout medium (SD/-Trp/-Leu/-His/-Ade). N/A indicates that an interaction could not be determined due to self-activating bait. (B) Rad16 truncations used in the immunoprecipitation analyses shown in (C). FL denotes the full-length protein. (C) The N-terminal 451 amino acids of Rad16 are both necessary and sufficient for co-immunoprecipitation (co-IP) with Pxd1. The co-IP between Rad16 and Pxd1 was performed in a *swi10Δ* background (DY16619). The N-terminal region of Pxd1 is prone to be cleaved off by proteolysis. (D) Cross-linking mass spectrometry (CXMS) analysis detected cross-links between Cdc24 and Pxd1 and between Cdc24 and Dna2. Only intermolecular cross-links are shown. (E) Cdc24 truncations used in the immunoprecipitation analyses shown in (F). FL denotes the full-length protein. (F) Amino acids 80–245 of Cdc24 are sufficient for co-IP with Pxd1.
(TIF)

Figure S2 **HO-based SSA assay.** (A) HO induction in the SSA strains resulted in a growth delay of *rad16Δ* and *swi10Δ*, but not wild-type cells. (B) HO induction in the SSA strains resulted in the loss of a *leu1^+^* marker residing between the two direct repeats. (C) A higher level of Rad52 foci was induced by HO in *pxd1Δ* than in wild-type cells during SSA repair. (D) Quantitation of the Rad52 foci at different time points after HO induction.
(TIF)

Figure S3 **Mating-type switching process and the iodine staining assay.** (A) Schematic depicting the mating-type switching process. An *M*-to-*P* switching event is shown as an example. H1 and H2 are homologous sequences flanking the *mat1* cassette and the two donor cassettes, *mat2-P* and *mat3-M*. The role of Rad16-Swi10 is believed to be removing the sequence beyond the H2 box on the newly synthesized strand after it extends outside of the donor cassette. (B) Schematics depicting the iodine staining assay used to determine the efficiency of mating-type switching. On a mating- and sporulation-compatible growth medium, wild-type heterothallic *h^90^* cells constantly switch mating type and thus can efficiently mate with each other to form iodine-stainable spores, whereas switching-defective mutant cells are mostly surrounded by cells of the same mating type, and thus only form spores at rare locations where cells of opposite mating types make contact.
(TIF)

Figure S4 **Synthetic lethality/sickness of** ***tdp1Δ swi10Δ*** **and** ***tdp1Δ pxd1Δ*** **.** (A) *swi10Δ* is synthetic lethal/sick with *tdp1Δ*, and this synthetic lethality/sickness can be rescued by *top1Δ*. Representative tetrads from a cross between a *swi10Δ* strain and a *tdp1Δ top1Δ* double mutant strain are shown. (B) The C-terminal region of Pxd1 is not required for rescuing of the synthetic lethality/sickness between *tdp1Δ* and *pxd1Δ*. Shown are representative tetrads from a cross between a *pxd1Δ* strain transformed with a plasmid expressing C-terminal-region–deleted Pxd1 and a *top1Δ tdp1Δ* strain. The plasmid was integrated at the *pxd1* locus. (C) A model for the two parallel pathways that can remove Top1cc. Pxd1 acts together with Rad16-Swi10 in a pathway redundant with a Tdp1-mediated pathway.
(TIF)

Figure S5 **Pxd1 activates Rad16-Swi10 and shows resemblance to SLX4.** (A) Rad16-Swi10 displays nuclease activity toward 3′ overhang and Y fork DNA but not 5′ overhang DNA. The Rad16-TAP immunoprecipitates were incubated separately with different substrates for 1 h. The reaction products were stained by ethidium bromide (EB) after separating by a 10% native PAGE gel. (B) Pxd1 is required for the efficient nuclease activity of Rad16-Swi10. The Rad16-TAP immunoprecipitates were incubated with 3′ overhang DNA for 1 h. The reaction products were analyzed as in (A). (C) The expression level and stability of Rad16-TAP is the same for the three strains used in (A), (B), and Figure 5A. Coomassie staining of PVDF membrane after immunodetection was used to control for protein loading and blotting efficiency. (D) Loss of Slx4 does not affect SSA repair. SSA assay was performed as in Figure 3B. (E) The middle region of Pxd1 shares sequence similarity with the MLR regions in metazoan Slx4 proteins. Red numbers indicate the number of residues omitted due to the lack of conservation. A155 in Pxd1 is denoted by an asterisk. Protein sequence accession numbers are NP_588130.1 (*Schizosaccharomyces pombe*), EPY50628.1 (*Schizosaccharomyces cryophilus*), EPX70982.1 (*Schizosaccharomyces octosporus*), NP_115820.2 (*Homo sapiens*), XP_414962.4 (*Gallus gallus*), XP_003201146.2 (*Danio rerio*), XP_002585778.1 (*Branchiostoma floridae*), XP_003728986.1 (*Strongylocentrotus purpuratus*), XP_001639182.1 (*Nematostella vectensis*), NP_648104.2 (*Drosophila melanogaster*), EFA07432.1 (*Tribolium castaneum*), EHJ68472.1 (*Danaus plexippus*), and XP_002408224.1 (*Ixodes scapularis*).
(TIF)

Figure S6 **Pxd1 C-terminal region binds to and antagonizes Dna2.** (A) The growth inhibition caused by Pxd1(227–351) overexpression can be suppressed by *pfh1-R20* and *pfh1-R23* mutations. (B) The 5A mutation weakened the interaction between Dna2-Cdc24 and Pxd1. The five mutated residues are labeled by asterisks in the sequence alignment. Dna2 and Cdc24-YFH co-overexpressed and purified from *pxd1Δ* cells were incubated with Smt3-Pxd1(227–351) or Smt3-Pxd1(227–351)-5A purified from *E. coli* for 2 h before immunoprecipitation with anti-YFP beads. The precipitates were washed and analyzed using immunoblotting with the indicated antibodies. (C) The expression levels and stability of Dna2 and Cdc24 are not significantly affected by the loss of Pxd1. Coomassie staining of PVDF membrane after immunodetection was used to control for protein loading and blotting efficiency. (D) The DNA damage sensitivity of *exo1Δ* is not altered by deleting *pxd1* or removing the C-terminal region of Pxd1.
(TIF)

Figure S7 **Multiple sequence alignment of Saw1 proteins.** The alignment was generated by MAFFT-L-INS-i (http://mafft.cbrc.jp/alignment/server/) and visualized with Jalview. The arrowhead points to the R19 residue in *S. cerevisiae* Saw1, which is important for the interaction between Saw1 and Rad1 [11]. The red bar denotes amino acids 244–250 in *S. cerevisiae* Saw1, which are important for DNA binding [11]. Protein sequence accession numbers are gi|6319292 (*Saccharomyces cerevisiae*), gi|366994494 (*Naumovozyma castellii*), gi|403215729 (*Kazachstania naganishii*), gi|50288411 (*Candida glabrata*), gi|367006196 (*Tetrapisispora phaffii*), gi|302307731 (*Ashbya gossypii*), gi|448106833 (*Millerozyma farinosa*), gi|50419339 (*Debaryomyces hansenii*), gi|260940873 (*Clavispora lusitaniae*), gi|562976212 (*Ogataea parapolymorpha*), gi|254567187 (*Komagataella pastoris*), gi|255730106 (*Candida tropicalis*), gi|528064676 (*Schizosaccharomyces octosporus*), and gi|19112258 (*Schizosaccharomyces pombe*).
(TIF)

Table S1 **Strains used in this study.**
(DOC)

Table S2 **Plasmids used in this study.**
(DOC)

Table S3 **Real-time PCR primers used in this study.**
(DOC)

Table S4 **Data used to generate the histograms in** **Figure 7** **.**
(XLS)
