# Supplementary figures and images for: Fission Yeast Pxd1 Promotes Proper DNA Repair by Activating Rad16XPF and Inhibiting Dna2
Source: PLoS Biol. 2014 Sep 9;12(9):e1001946. doi: 10.1371/journal.pbio.1001946 (PMC4159138; doi:10.1371/journal.pbio.1001946)

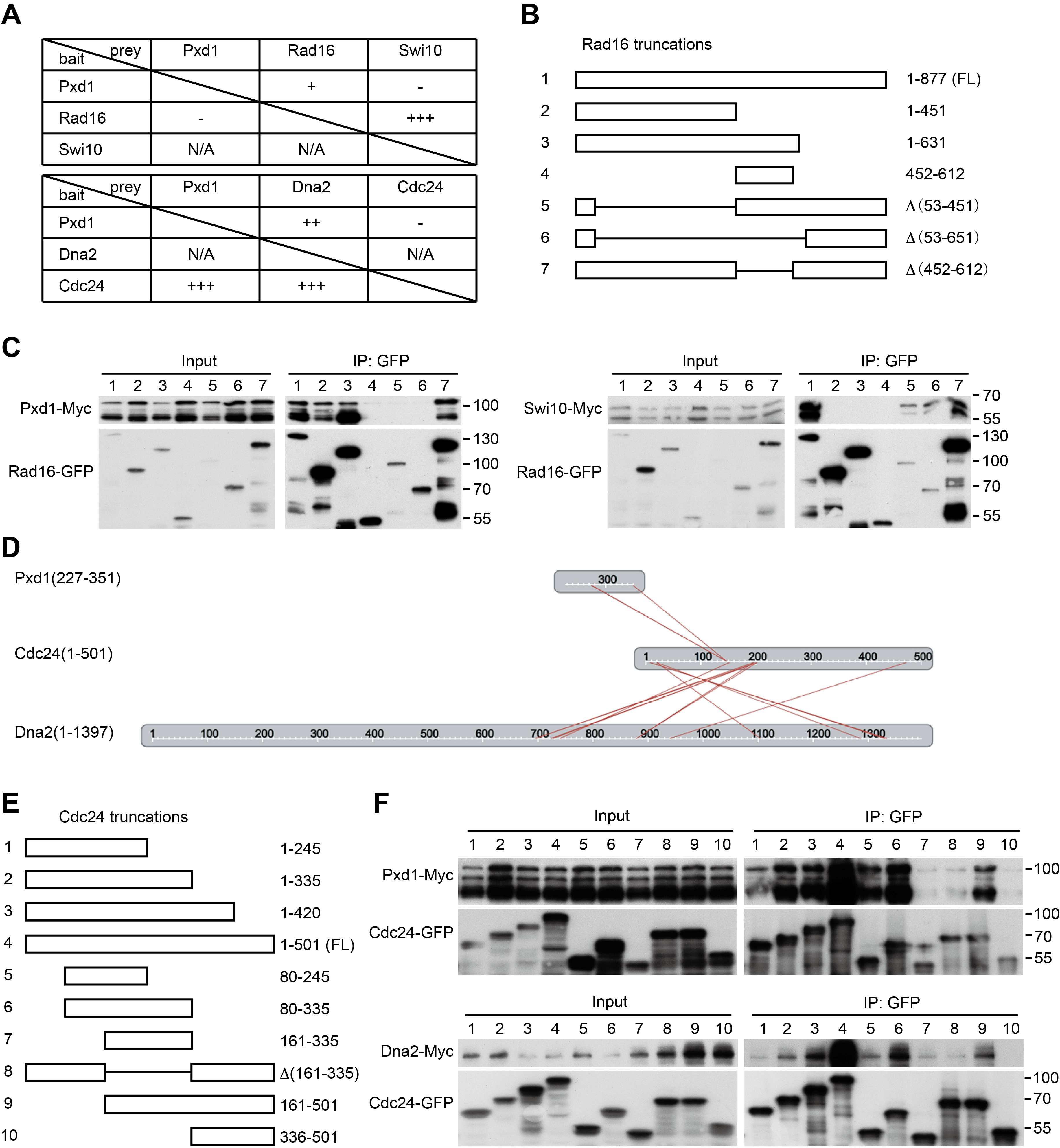

Supplement: Figure S1 — Mapping the binding interfaces on the Pxd1-binding proteins. (A) In yeast two-hybrid assays, Pxd1 interacts with Rad16 and Dna2 as a bait and interacts with Cdc24 as a prey. The interactions were scored according to the growth on the quadruple dropout medium (SD/-Trp/-Leu/-His/-Ade). N/A indicates that an interaction could not be determined due to self-activating bait. (B) Rad16 truncations used in the immunoprecipitation analyses shown in (C). FL denotes the full-length protein. (C) The N-terminal 451 amino acids of Rad16 are both necessary and sufficient for co-immunoprecipitation (co-IP) with Pxd1. The co-IP between Rad16 and Pxd1 was performed in a swi10Δ background (DY16619). The N-terminal region of Pxd1 is prone to be cleaved off by proteolysis. (D) Cross-linking mass spectrometry (CXMS) analysis detected cross-links between Cdc24 and Pxd1 and between Cdc24 and Dna2. Only intermolecular cross-links are shown. (E) Cdc24 truncations used in the immunoprecipitation analyses shown in (F). FL denotes the full-length protein. (F) Amino acids 80–245 of Cdc24 are sufficient for co-IP with Pxd1. (TIF) [file pbio.1001946.s001.tif]

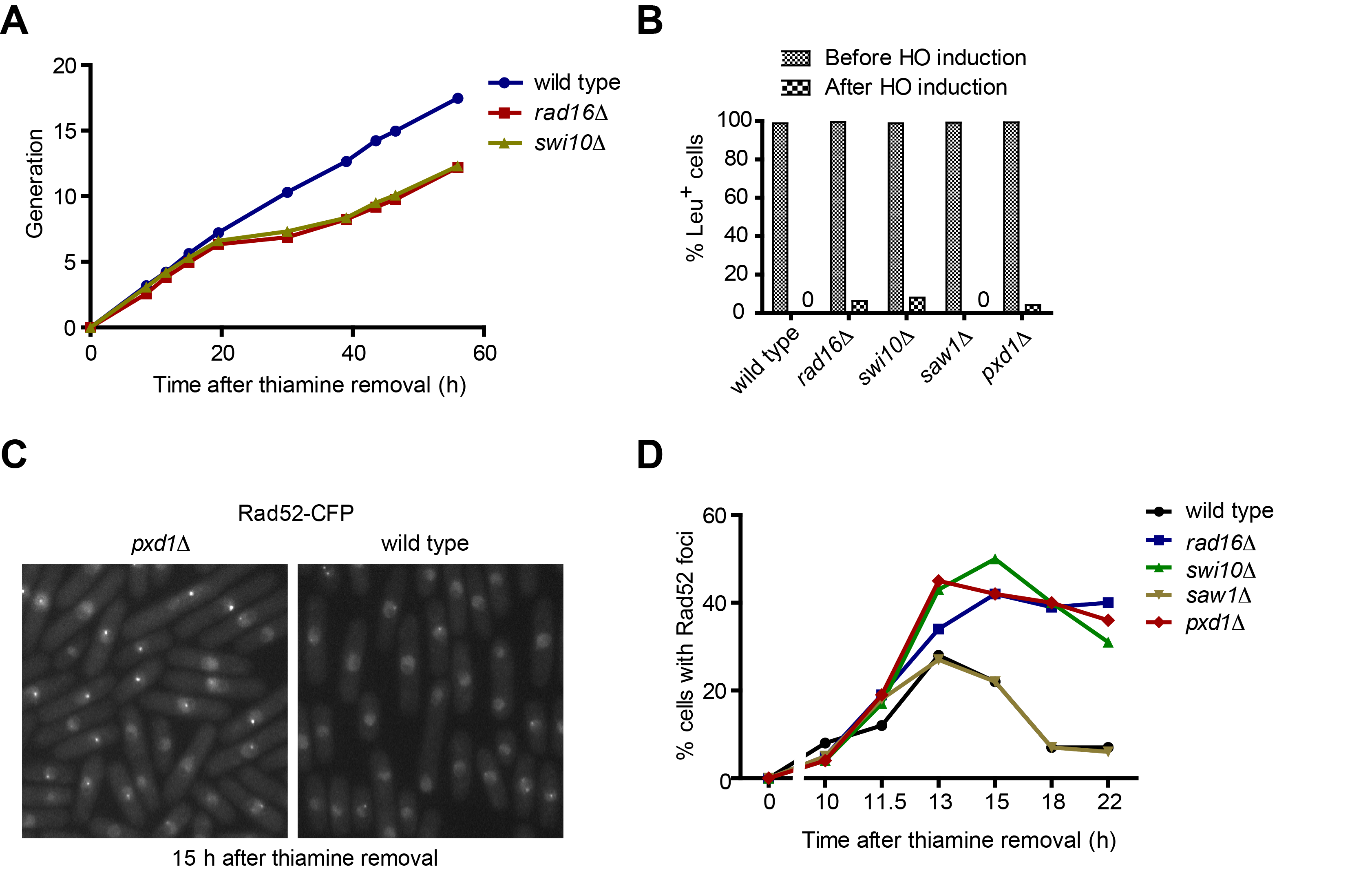

Supplement: Figure S2 — HO-based SSA assay. (A) HO induction in the SSA strains resulted in a growth delay of rad16Δ and swi10Δ, but not wild-type cells. (B) HO induction in the SSA strains resulted in the loss of a leu1+ marker residing between the two direct repeats. (C) A higher level of Rad52 foci was induced by HO in pxd1Δ than in wild-type cells during SSA repair. (D) Quantitation of the Rad52 foci at different time points after HO induction. (TIF) [file pbio.1001946.s002.tif]

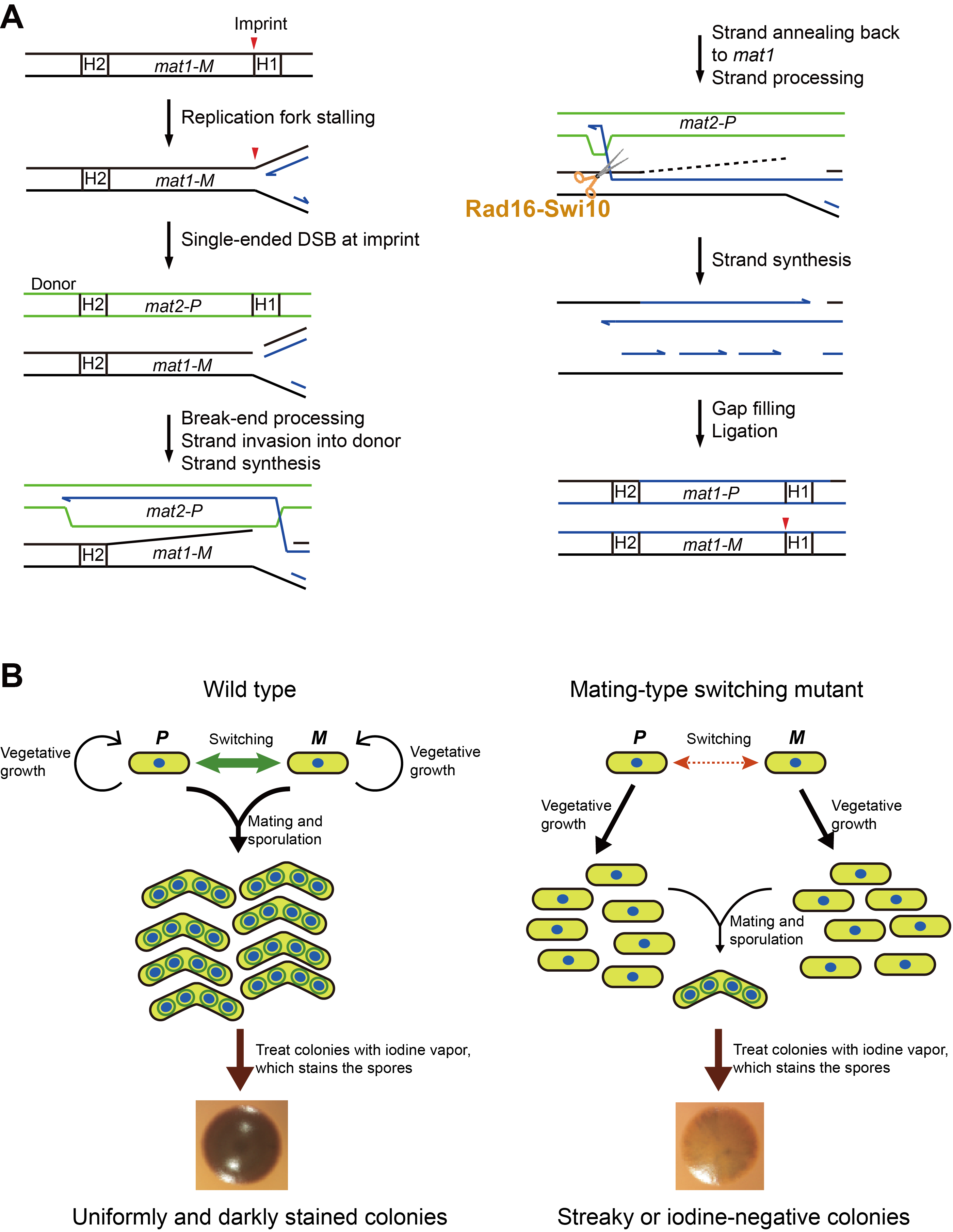

Supplement: Figure S3 — Mating-type switching process and the iodine staining assay. (A) Schematic depicting the mating-type switching process. An M-to-P switching event is shown as an example. H1 and H2 are homologous sequences flanking the mat1 cassette and the two donor cassettes, mat2-P and mat3-M. The role of Rad16-Swi10 is believed to be removing the sequence beyond the H2 box on the newly synthesized strand after it extends outside of the donor cassette. (B) Schematics depicting the iodine staining assay used to determine the efficiency of mating-type switching. On a mating- and sporulation-compatible growth medium, wild-type heterothallic h90 cells constantly switch mating type and thus can efficiently mate with each other to form iodine-stainable spores, whereas switching-defective mutant cells are mostly surrounded by cells of the same mating type, and thus only form spores at rare locations where cells of opposite mating types make contact. (TIF) [file pbio.1001946.s003.tif]

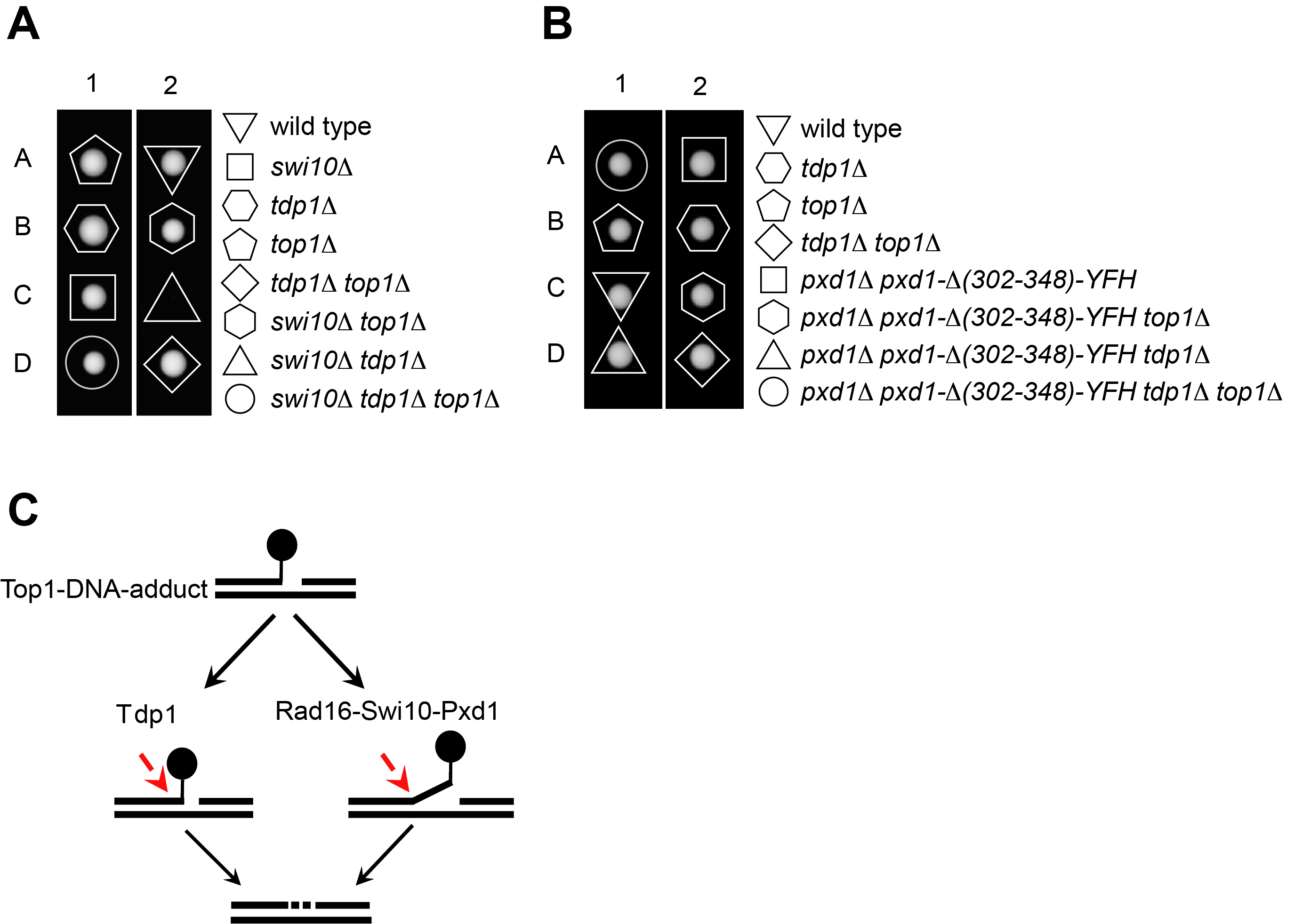

Supplement: Figure S4 — Synthetic lethality/sickness of tdp1Δ swi10Δ and tdp1Δ pxd1Δ . (A) swi10Δ is synthetic lethal/sick with tdp1Δ, and this synthetic lethality/sickness can be rescued by top1Δ. Representative tetrads from a cross between a swi10Δ strain and a tdp1Δ top1Δ double mutant strain are shown. (B) The C-terminal region of Pxd1 is not required for rescuing of the synthetic lethality/sickness between tdp1Δ and pxd1Δ. Shown are representative tetrads from a cross between a pxd1Δ strain transformed with a plasmid expressing C-terminal-region–deleted Pxd1 and a top1Δ tdp1Δ strain. The plasmid was integrated at the pxd1 locus. (C) A model for the two parallel pathways that can remove Top1cc. Pxd1 acts together with Rad16-Swi10 in a pathway redundant with a Tdp1-mediated pathway. (TIF) [file pbio.1001946.s004.tif]

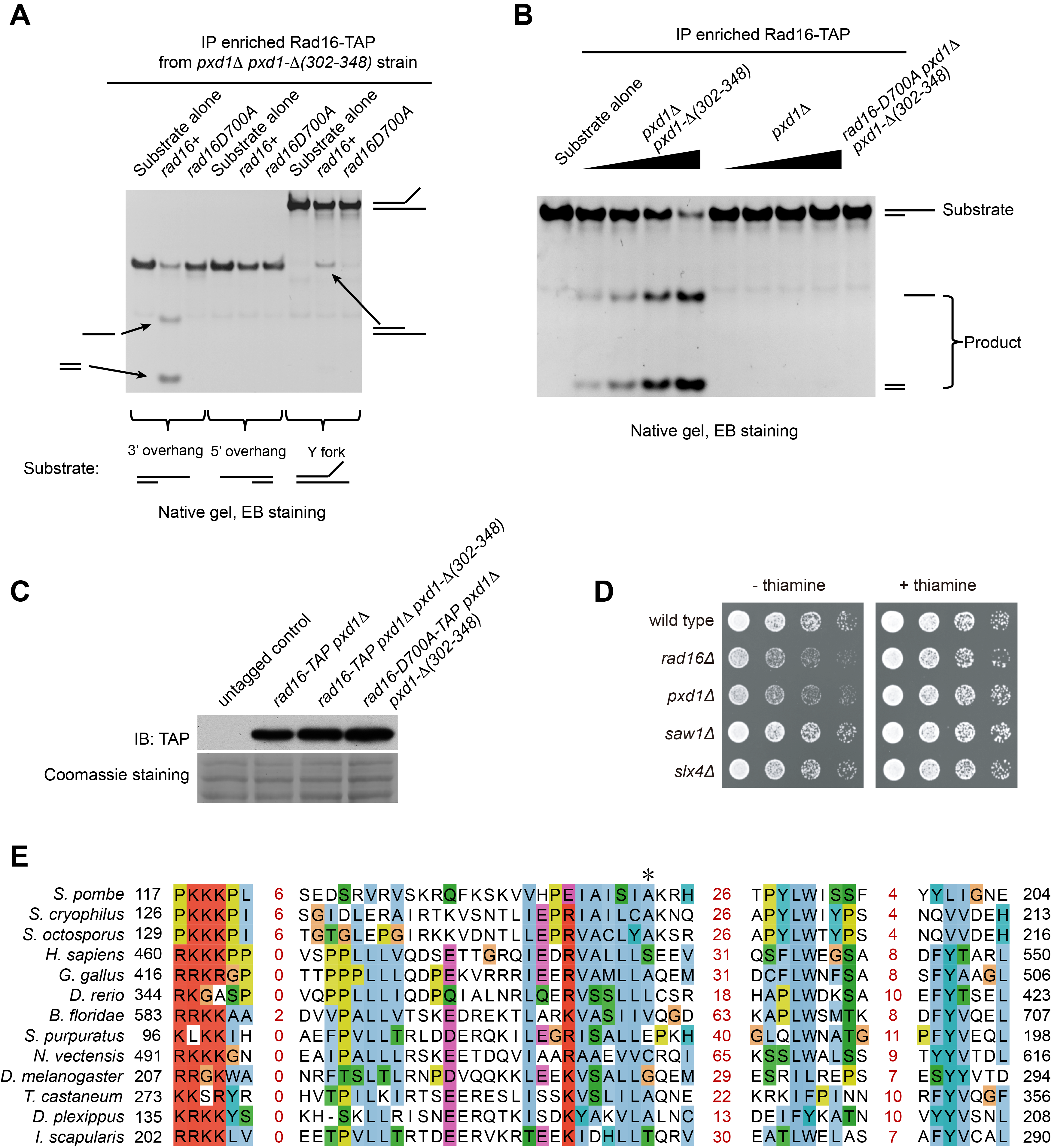

Supplement: Figure S5 — Pxd1 activates Rad16-Swi10 and shows resemblance to SLX4. (A) Rad16-Swi10 displays nuclease activity toward 3′ overhang and Y fork DNA but not 5′ overhang DNA. The Rad16-TAP immunoprecipitates were incubated separately with different substrates for 1 h. The reaction products were stained by ethidium bromide (EB) after separating by a 10% native PAGE gel. (B) Pxd1 is required for the efficient nuclease activity of Rad16-Swi10. The Rad16-TAP immunoprecipitates were incubated with 3′ overhang DNA for 1 h. The reaction products were analyzed as in (A). (C) The expression level and stability of Rad16-TAP is the same for the three strains used in (A), (B), and Figure 5A. Coomassie staining of PVDF membrane after immunodetection was used to control for protein loading and blotting efficiency. (D) Loss of Slx4 does not affect SSA repair. SSA assay was performed as in Figure 3B. (E) The middle region of Pxd1 shares sequence similarity with the MLR regions in metazoan Slx4 proteins. Red numbers indicate the number of residues omitted due to the lack of conservation. A155 in Pxd1 is denoted by an asterisk. Protein sequence accession numbers are NP_588130.1 (Schizosaccharomyces pombe), EPY50628.1 (Schizosaccharomyces cryophilus), EPX70982.1 (Schizosaccharomyces octosporus), NP_115820.2 (Homo sapiens), XP_414962.4 (Gallus gallus), XP_003201146.2 (Danio rerio), XP_002585778.1 (Branchiostoma floridae), XP_003728986.1 (Strongylocentrotus purpuratus), XP_001639182.1 (Nematostella vectensis), NP_648104.2 (Drosophila melanogaster), EFA07432.1 (Tribolium castaneum), EHJ68472.1 (Danaus plexippus), and XP_002408224.1 (Ixodes scapularis). (TIF) [file pbio.1001946.s005.tif]

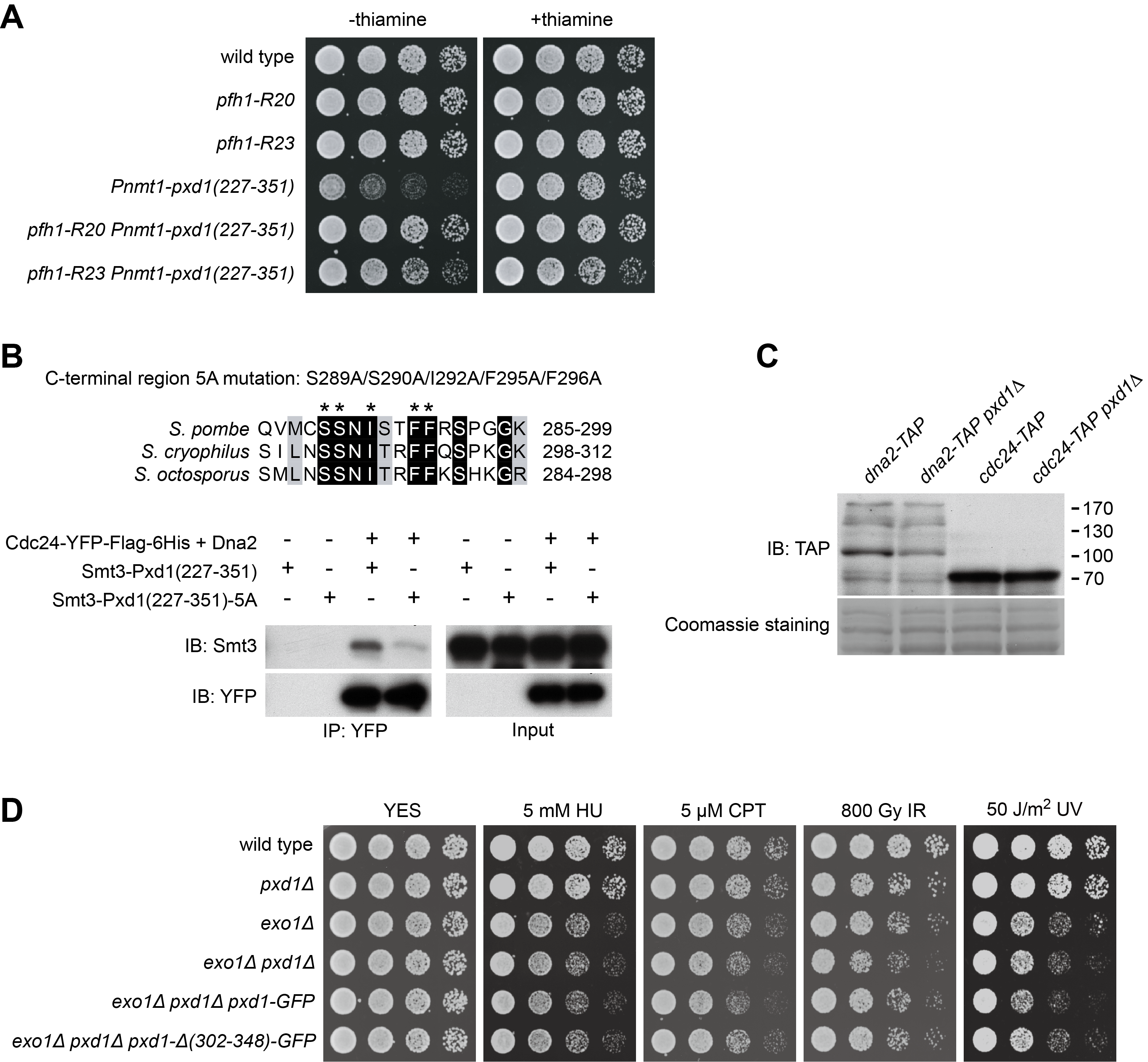

Supplement: Figure S6 — Pxd1 C-terminal region binds to and antagonizes Dna2. (A) The growth inhibition caused by Pxd1(227–351) overexpression can be suppressed by pfh1-R20 and pfh1-R23 mutations. (B) The 5A mutation weakened the interaction between Dna2-Cdc24 and Pxd1. The five mutated residues are labeled by asterisks in the sequence alignment. Dna2 and Cdc24-YFH co-overexpressed and purified from pxd1Δ cells were incubated with Smt3-Pxd1(227–351) or Smt3-Pxd1(227–351)-5A purified from E. coli for 2 h before immunoprecipitation with anti-YFP beads. The precipitates were washed and analyzed using immunoblotting with the indicated antibodies. (C) The expression levels and stability of Dna2 and Cdc24 are not significantly affected by the loss of Pxd1. Coomassie staining of PVDF membrane after immunodetection was used to control for protein loading and blotting efficiency. (D) The DNA damage sensitivity of exo1Δ is not altered by deleting pxd1 or removing the C-terminal region of Pxd1. (TIF) [file pbio.1001946.s006.tif]

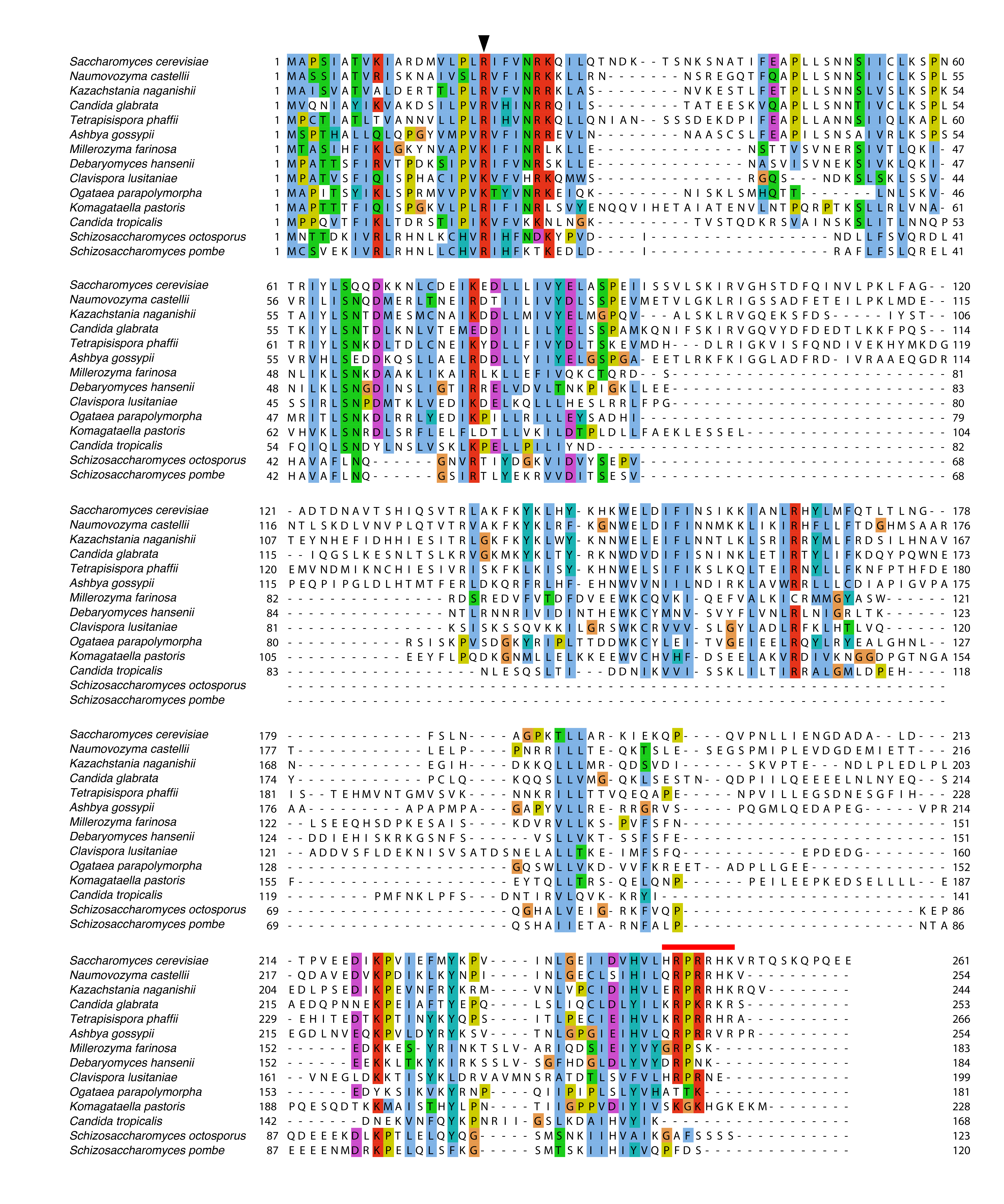

Supplement: Figure S7 — Multiple sequence alignment of Saw1 proteins. The alignment was generated by MAFFT-L-INS-i (http://mafft.cbrc.jp/alignment/server/) and visualized with Jalview. The arrowhead points to the R19 residue in S. cerevisiae Saw1, which is important for the interaction between Saw1 and Rad1 [11]. The red bar denotes amino acids 244–250 in S. cerevisiae Saw1, which are important for DNA binding [11]. Protein sequence accession numbers are gi|6319292 (Saccharomyces cerevisiae), gi|366994494 (Naumovozyma castellii), gi|403215729 (Kazachstania naganishii), gi|50288411 (Candida glabrata), gi|367006196 (Tetrapisispora phaffii), gi|302307731 (Ashbya gossypii), gi|448106833 (Millerozyma farinosa), gi|50419339 (Debaryomyces hansenii), gi|260940873 (Clavispora lusitaniae), gi|562976212 (Ogataea parapolymorpha), gi|254567187 (Komagataella pastoris), gi|255730106 (Candida tropicalis), gi|528064676 (Schizosaccharomyces octosporus), and gi|19112258 (Schizosaccharomyces pombe). (TIF) [file pbio.1001946.s007.tif]
