# Supplementary material for: Fission Yeast Pxd1 Promotes Proper DNA Repair by Activating Rad16XPF and Inhibiting Dna2
Source: PLoS Biol. 2014 Sep 9;12(9):e1001946. doi: 10.1371/journal.pbio.1001946 (PMC4159138; doi:10.1371/journal.pbio.1001946)
Supplement: Table S1 — Strains used in this study. (DOC) [file pbio.1001946.s008.doc]

**Table S1**. Strains used in this study

| Strain | Mating  Type | Genotype |
| --- | --- | --- |
| DY1948 | h+ | *saw1-YFH::leu1+* |
| DY6285 | h+ | *his3-D1 pxd1Δ::natMX leu1-32::pJK148-pxd1-YFH(leu1+)* |
| DY5300 | h+ | *his3-D1 pxd1Δ::natMX rad16-13xMyc::kanMX leu1::pJK148-pxd1-TAP(leu1+)* |
| DY5389 | h+ | *his3-D1 pxd1Δ::natMX rad16-13xMyc::kanMX leu1-32::pJK148-pxd1-**Δ(9-100)-TAP(leu1+)* |
| DY5390 | h+ | *his3-D1 pxd1Δ::natMX rad16-13xMyc::kanMX leu1-32::pJK148-pxd1-Δ(9-226)-TAP(leu1+)* |
| DY5391 | h+ | *his3-D1 pxd1Δ::natMX rad16-13xMyc::kanMX leu1-32::pJK148-pxd1-Δ(234-351)-TAP(leu1+)* |
| DY5392 | h+ | *his3-D1 pxd1Δ::natMX rad16-13xMyc::kanMX leu1-32::pJK148-pxd1-Δ(108-226)-TAP(leu1+)* |
| DY5301 | h+ | *his3-D1 pxd1Δ::natMX cdc24-13xMyc::kanMX leu1::pJK148-pxd1-TAP(leu1+)* |
| DY5393 | h+ | *his3-D1 pxd1Δ::natMX cdc24-13xMyc::kanMX leu1::pJK148-pxd1-Δ(9-100)-TAP(leu1+)* |
| DY5394 | h+ | *his3-D1 pxd1Δ::natMX cdc24-13xMyc::kanMX leu1::pJK148-pxd1-Δ(9-226)-TAP(leu1+)* |
| DY5395 | h+ | *his3-D1 pxd1Δ::natMX cdc24-13xMyc::kanMX leu1::pJK148-pxd1-Δ(234-351)-TAP(leu1+)* |
| DY5396 | h+ | *his3-D1 pxd1Δ::natMX cdc24-13xMyc::kanMX leu1::pJK148-pxd1-Δ(108-226)-TAP(leu1+)* |
| DY4727 | h- | *leu1-32 his3-D1 cdc24-13xMyc::kanMX rad16-TAP::hphMX* |
| DY5180 | h- | *leu1-32 his3-D1 cdc24-13xMyc::kanMX rad16-TAP::hphMX pxd1Δ::natMX* |
| DY4707 | h- | *leu1-32 his3-D1 cdc24-13xMyc::kanMX saw1-TAP::hphMX* |
| DY5176 | h- | *leu1-32 his3-D1 cdc24-13xMyc::kanMX saw1-TAP::hphMX pxd1Δ::natMX* |
| DY16619 | h? | *leu1-32 his3-D1 pxd1-13xMyc::kanMX swi10Δ::natMX rad16Δ::natMX* |
| DY4310 | h+ | *leu1-32 his3-D1 swi10-13xMyc::kanMX* |
| DY7398 | h- | *leu1-32 his3-D1 pxd1-13xMyc::kanMX* |
| DY3374 | h+ | *leu1-32 his3-D1 dna2-13xMyc::kanMX* |
| LD328 | h+ | *leu1-32 his3-D1* |
| LD346 | h+ | *leu1-32 ura4-D18 chk1Δ::ura4+* |
| DY5949 | h+ | *leu1-32 his3-? rad16Δ::kanMX* |
| DY8448 | h+ | *leu1-32 his3-D1 swi10Δ::kanMX* |
| DY8472 | h+ | *leu1-32 his3-D1 rhp14Δ::kanMX* |
| DY3372 | h+ | *leu1-32 his3-D1 pxd1Δ::kanMX* |
| DY3373 | h+ | *leu1-32 his3-D1 saw1Δ::kanMX* |
| DY8710 | h+ | *leu1-32 his3-D1 rad16Δ::kanMX pxd1Δ::natMX* |
| DY8707 | h+ | *leu1-32 his3-D1 pxd1Δ::natMX rhp14Δ::kanMX* |
| DY8720 | h+ | *leu1-32 his3-D1 rad16Δ::kanMX pxd1Δ::natMX rhp14Δ::kanMX* |
| DY1012 | h- | *leu1-32 his3-D1 ura4-294 arg3::HOsite-natMX(arg3-) erg7ter::lacOrepeat(ura4+) ars1::pJR1-41XH+HO(his3+) lys1-131::Pdis1-mCherry-LacI(lys1+) rad22-2xCFP::hphMX* |
| DY2392 | h- | *cmb1+::pDB176(leu1+)* in DY1012 background |
| DY3328 | h+ | *his3-? ura4-? rad16Δ::kanMX* in DY2392 background |
| DY3329 | h+ | *ura4-? swi10Δ::kanMX* in DY2392 background |
| DY3471 | h+ | *ura4-? pxd1Δ::kanMX* in DY2392 background |
| DY3349 | h+ | *ura4-? saw1Δ::kanMX* in DY2392 background |
| DY3551 | h+ | *his3-? ura4-? rad16Δ::kanMX pxd1Δ::kanMX* in DY2392 background |
| DY3554 | h+ | *ura4-?*  *swi10Δ::kanMX pxd1Δ::kanMX* in DY2392 background |
| DY3560 | h+ | *ura4-?*  *saw1Δ::kanMX pxd1Δ::kanMX* in DY2392 background |
| DY5539 | h+ | *ura4-? pxd1Δ::kanMX leu1-32::pJK148-pxd1-TAP(leu1+)* in DY2392 background |
| DY5541 | h+ | *ura4-? pxd1Δ::kanMX leu1-32::pJK148-pxd1-Δ(9-100)-TAP(leu1+)*  in DY2392 background |
| DY5543 | h+ | *ura4-? pxd1Δ::kanMX leu1-32::pJK148-pxd1-Δ(9-226)-TAP(leu1+)*  in DY2392 background |
| DY5545 | h+ | *ura4-? pxd1Δ::kanMX leu1-32::pJK148-pxd1-Δ(234-351)-TAP(leu1+)*  in DY2392 background |
| DY5547 | h+ | *ura4-? pxd1Δ::kanMX*  *leu1-32::pJK148-pxd1-Δ(108-226)-TAP(leu1+)*  *in DY2392 background* |
| DY4840 | h+ | *leu1-32 his3-D1 ura4-294(or ura4-D18) arg3::HOsite-natMX(arg3-) erg7ter::lacOrepeat(ura4+) ars1::pJR1-41XH+HO(his3+) lys1-131::Pdis1-mCherry-LacI(lys1+) rad22-2xCFP::hphMX* |
| DY5999 | h+ | *cmb1+::pDB459(leu1+)* in DY4840 background |
| DY7800 | h+ | *ura4-? pxd1Δ::kanMX* in DY5999 background |
| DY7806 | h+ | *his3-? ura4-? swi10Δ::kanMX* in DY5999 background |
| DY7801 | h+ | *his3-? ura4-? saw1Δ::kanMX* in DY5999 background |
| DY3488 | h90 | *leu1-32 his2Δ::hphMX* |
| DY6285 | h+ | *leu1-32::pJK148-pxd1-YFH(leu1+) his3-D1 pxd1Δ::natMX* |
| DY6305 | h+ | *leu1-32::pJK148-pxd1-Δ(108-226)-YFH(leu1+)*  *his3-D1 pxd1Δ::natMX* |
| DY6301 | h+ | *leu1-32::pJK148-pxd1-Δ(234-351)-YFH(leu1+)*  *his3-D1 pxd1Δ::natMX* |
| DY4597 | h+ | *leu1-32 his3-D1 pxd1**Δ::natMX* |
| DY6067 | h- | *leu1-32 his3-D1 tdp1Δ::hphMX* |
| DY9452 | h+ | *leu1-32 his3-D1 psp3Δ::kanM isp6Δ::hphMX pxd1Δ::natMX rad16-TAP::hphMX* |
| DY9577 | h+ | *his3-D1 psp3Δ::kanMX isp6Δ::hphMX pxd1Δ::natMX rad16-TAP::hphMX leu1-32::pJK148-pxd1-Δ(302-348)-YFH(leu1+)* |
| DY9578 | h+ | *his3-D1 psp3Δ::kanMX,isp6Δ::hphMX pxd1Δ::natMX rad16-D700A-TAP::hphMX leu1-32::pJK148-pxd1-Δ(302-348)-YFH(leu1+)* |
| DY8274 | h+ | *his3-D1 psp3Δ::kanMX isp6Δ::hphMX pxd1Δ::natMX leu1-32::pDUAL-rad16-YFH-swi10(leu1+)* |
| DY8734 | h+ | *ura4-? pxd1Δ::kanMX leu1-32::pJK148-pxd1-YFH(leu1+)*  in DY5999 background |
| DY10813 | h+ | *ura4-? pxd1Δ::kanMX*  *leu1-32::pJK148-pxd1-A155D/E172A-YFH(leu1+)*  in DY5999 background |
| DY13632 | h+ | *his3-D1 psp3Δ::kanMX isp6Δ::hphMX pxd1Δ::natMX rad16-TAP::hphMX*  *leu1-32::pJK148-pxd1-Δ(302-348)-13xmyc(leu1+)* |
| DY13633 | h+ | *his3-D1 psp3Δ::kanMX isp6Δ::hphMX pxd1Δ::natMX rad16-TAP::hphMX leu1-32::pJK148-pxd1-Δ(302-348)-A155D/E172A-13xmyc(leu1+)* |
| DY3305 | h+ | *pxd1Δ::kanMX* in DY4840 background |
| DY3837 | h+ | *rqh1Δ::kanMX* in DY4840 background |
| DY12147 | h+ | *his3-? ura4? rqh1Δ::kanMX pxd1Δ::natMX* in DY4840 background |
| DY8023 | h+ | *his3-? exo1Δ::ura4+* in DY4840 background |
| DY8024 | h+ | *his3-? ura4-? exo1Δ::ura4+ pxd1Δ::natMX* in DY4840 background |
| DY12137 | h- | *his3-? ura4-? rqh1Δ::kanMX exo1Δ::ura4+* in DY4840 background |
| DY12143 | h+ | *his3-? ura4-? rqh1Δ::kanMX exo1::ura4+ pxd1Δ::natMX* in DY4840 background |
| DY12097 | h+ | *his3-? ura4-? exo1Δ::ura4+ pxd1Δ::natMX*  *leu1-32::pJK148-pxd1-YFH(leu1+)* in DY4840 background |
| DY12100 | h+ | *his3-? ura4-? exo1Δ::ura4 pxd1Δ::natMX leu1-32::pJK148-pxd1-Δ(9-100)-YFH(leu1+)*  in DY4840 background |
| DY12109 | h+ | *his3-? ura4-? exo1Δ::ura4 pxd1Δ::natMX leu1-32::pJK148-pxd1-Δ(108-226)-YFH(leu1+)*  in DY4840 background |
| DY12112 | h+ | *his3-? ura4-? exo1Δ::ura4 pxd1Δ::natMX leu1-32::pJK148-pxd1-Δ(302-348)-YFH(leu1+)*  in DY4840 background |
| DY12103 | h+ | *his3-? ura4-? exo1Δ::ura4 pxd1Δ::natMX leu1-32::pJK148-pxd1-Δ(9-226)-YFH(leu1+)*  in DY4840 background |
| DY8272 | h+ | *his3-D1 psp3Δ::kanMX isp6Δ::hphMX pxd1Δ::natMX leu1-32::pDUAL-cdc24-YFH+dna2(leu1+)* |
| DY16171 | h+ | *his3-? leu1-32::pDUAL-pxd1(227-351)-TAP(leu1+)* |
| DY16157 | h+ | *his3-D1 pxd1Δ::natMX leu1-32::pDUAL-pxd1(227-351)-TAP(leu1+)* |
| DY16159 | h+ | *his3-D1 pxd1Δ::natMX leu1-32::pDUAL-pxd1(227-351)-5A-TAP(leu1+)* |
| DY3606 | h- | *leu1-32 pfh1-R20* |
| DY3607 | h- | *leu1-32 pfh1-R23* |
| DY16168 | h+ | *his3-? leu1-32::pDUAL-pxd1(227-351)-TAP(leu1+) pfh1-R20* |
| DY16170 | h+ | *his3-? leu1-32::pDUAL-pxd1(227-351)-TAP(leu1+) pfh1-R23* |
| DY16174 | h+ | *his3-D1 ura4-? leu1-32::pDUAL-pxd1(227-351)-TAP(leu1+) ars1::pDUAL+cdc24-YFH+dna2(ura4+)* |
| DY16175 | h- | *his3-D1 ura4-? leu1-32 ars1::pDUAL+cdc24-YFH+dna2(ura4+)* |
| DY16178 | h+ | *his3-D1 ura4-? leu1-32::pDUAL-pxd1(227-351)-TAP(leu1+) ars1::pDUAL+dna2-YFH(ura4+)* |
| DY16179 | h- | *his3-D1 ura4-? leu1-32 ars1::pDUAL+dna2-YFH(ura4+)* |
| DY12070 | h+ | *leu1-32 his3-D1 ura4-? arg3::HOsite-natMX(arg3-) erg7ter::lacOrepeat(ura4+) ars1::pJR1-41XH+HO(his3+) lys1-131::Pdis1-mCherry-LacI(lys1+) rad22-2xCFP::hphMX cmb1+::pDB1637(leu1+)* |
| DY12074 | h+ | *exo1Δ::ura4+* in DY12070 background |
| DY12079 | h+ | *pxd1::kanMX::pDB1632(ura4+)* in DY12070 background |
| DY12084 | h+ | *exo1Δ::ura4+* *pxd1::kanMX::pDB1632(ura4+)* in DY12070 background |
