# Supplementary material for: Fission Yeast Pxd1 Promotes Proper DNA Repair by Activating Rad16XPF and Inhibiting Dna2
Source: PLoS Biol. 2014 Sep 9;12(9):e1001946. doi: 10.1371/journal.pbio.1001946 (PMC4159138; doi:10.1371/journal.pbio.1001946)
Supplement: Table S2 — Plasmids used in this study. (DOC) [file pbio.1001946.s009.doc]

**Table S2. Plasmids** used in this study

| Plasmid | Description |
| --- | --- |
| pDB448 | pJK148+pxd1-TAP |
| pDB508 | pJK148+pxd1-Δ(9-100)-TAP |
| pDB510 | pJK148+pxd1-Δ(9-226)-TAP |
| pDB512 | pJK148+pxd1-Δ(234-351)-TAP |
| pDB514 | pJK148+pxd1-Δ(108-226)-TAP |
| pDB2364 | Y2H-prey-vector+cdc24 |
| pDB2366 | Y2H-bait-vector+cdc24 |
| pDB2371 | Y2H-prey-vector+dna2 |
| pDB2372 | Y2H-bait-vector+dna2 |
| pDB2373 | Y2H-prey-vector+pxd1 |
| pDB2375 | Y2H-bait-vector+pxd1 |
| pDB2455 | Y2H-prey-vector+rad16 |
| pDB2457 | Y2H-bait-vector+rad16 |
| pDB2458 | Y2H-prey-vector+swi10 |
| pDB2459 | Y2H-bait-vector+swi10 |
| pDB2701 | pDUAL+Pnmt41-rad16(1-451)-GFP |
| pDB2703 | pDUAL+Pnmt41-rad16(1-631)-GFP |
| pDB2704 | pDUAL+Pnmt41-rad16(452-612)-GFP |
| pDB2706 | pDUAL+Pnmt41-rad16(1-877)-GFP |
| pDB2708 | pDUAL+Pnmt41-rad16-Δ(53-451)-GFP |
| pDB2710 | pDUAL+Pnmt41-rad16-Δ(53-651)-GFP |
| pDB2712 | pDUAL+Pnmt41-rad16-Δ(452-612)-GFP |
| pDB2509 | pDUAL+Pnmt41-cdc24(1-245)-GFP |
| pDB2511 | pDUAL+Pnmt41-cdc24(1-335)-GFP |
| pDB2512 | pDUAL+Pnmt41-cdc24(1-420)-GFP |
| pDB2513 | pDUAL+Pnmt41-cdc24(1-501)-GFP |
| pDB2515 | pDUAL+Pnmt41-cdc24(80-245)-GFP |
| pDB2517 | pDUAL+Pnmt41-cdc24(80-335)-GFP |
| pDB2519 | pDUAL+Pnmt41-cdc24(161-335)-GFP |
| pDB2520 | pDUAL+Pnmt41-cdc24-Δ(161-335)-GFP |
| pDB2522 | pDUAL+Pnmt41-cdc24(161-501)-GFP |
| pDB2524 | pDUAL+Pnmt41-cdc24(336-501)-GFP |
| pDB169 | pJK148+arg3-upstream |
| pDB174 | pJK148+arg3-upstream+cmb1 |
| pDB176 | pJK148+arg3-upstream+cmb1+cmb1-downstream |
| pDB459 | pJK148+arg3-upstream+BstUI+cmb1-downsteam |
| pDB454 | pJK148+Ppxd1-pxd1-YFH |
| pDB516 | pJK148+Ppxd1-pxd1-Δ(9-100)-YFH |
| pDB518 | pJK148+Ppxd1-pxd1-Δ(9-226)-YFH |
| pDB519 | pJK148+Ppxd1-pxd1-Δ(234-351)-YFH |
| pDB521 | pJK148+Ppxd1-pxd1-Δ(108-226)-YFH |
| pDB527 | pJK148+Ppxd1-pxd1-Δ(302-348)-YFH |
| pDB916 | pJK148+Ppxd1-pxd1-Δ(302-348)-A155D/E172A-YFH |
| pDB1295 | pDUAL+Pnmt1-rad16-YFH+Pnmt1-swi10 |
| pDB1297 | pDUAL+Pnmt1-cdc24-YFH+Pnmt1-dna2 |
| pDB2217 | pETDuet+smt3-pxd1(101-351)-6*His |
| pDB2219 | pETDuet+smt3-pxd1(101-351)-A155D/E172A-6*His |
| pDB1832 | pETDuet+6*His-smt3-pxd1(227-351) |
| pDB1834 | pETDuet+6*His-smt3-pxd1(227-351)-5A |
| pDB2129 | pETDuet+6*His-GST-Rpa1+Rpa3 |
| pDB2127 | pCDFDuet-RPA2 |
| pDB2663 | pDUAL+Pnmt1-pxd1(227-351)-TAP |
| pDB2664 | pDUAL+Pnmt1-pxd1(227-351)-5A-TAP |
| pDB2690 | pDUAL+Pnmt1-dna2-YFH |
| pDB1637 | pJK148+arg3-upstream+cmb1+cmb1-downstream+donor1 |
| pDB1632 | pJK148+Ppxd1-pxd1-Δ(302-348)-YFH+ura4 |
