# Supplementary material for: Fission Yeast Pxd1 Promotes Proper DNA Repair by Activating Rad16XPF and Inhibiting Dna2
Source: PLoS Biol. 2014 Sep 9;12(9):e1001946. doi: 10.1371/journal.pbio.1001946 (PMC4159138; doi:10.1371/journal.pbio.1001946)
Supplement: Table S3 — Real-time PCR primers used in this study. (DOC) [file pbio.1001946.s010.doc]

**Table S3. Realtime PCR primers** used in this study

| Name | Sequence | Description |
| --- | --- | --- |
| LD252 | TGCTCCTCCTGAGCGTAAATACTCTGTCTG | Actin control |
| LD253 | AACGATACCAGGTCCGCTCTCATCATACTC | Actin control |
| HO_cut_F | ACTCACTATAGGGCGAATTGGGTACG | HO cut efficiency |
| HO_cut_R | GCGCCTTAATTAACCCGGTACAGT | HO cut efficiency |
| Oligo407 | CCAGCTGATATTCGCAAAGTACTGGGA | ssDNA tail cleavage |
| Oligo408 | TCTCACGTTTGCAGCACTTTCATGT | ssDNA tail cleavage |
| 3A | TGTTGCGGAAAGCTGAAAGGTACCTG | DNA resection at 35 bp |
| 3B | CTCGCAGTCTGAGAGAGAACTAGATATCGG | DNA resection at 35 bp |
| 4A | CATAAGGTTTGCATACACCGTTGGGTAGG | DNA resection at 3.1 kb |
| 4B | CGGAAAGAACTTGATTGGATTGATTAACACTCATCC | DNA resection at 3.1 kb |
| 6A | tagcaccggctcgtctattt | DNA resection at 5.4 kb |
| 6B | AAGCAATGGGACTTCAATCG | DNA resection at 5.4 kb |
| 7A | TCAAAGCTGCGAAACAACAC | DNA resection at 9.4 kb |
| 7B | TCGGTGCAGACGATCAATAA | DNA resection at 9.4 kb |
| 8A | AGCAGGAGAGAGATATGGAACT | DNA resection at 20 kb |
| 8B | GGGAAGTCTCCTACTTCAACTT | DNA resection at 20 kb |
